# Supplementary material for: The E3 ligase TRIM1 ubiquitinates LRRK2 and controls its localization, degradation, and toxicity
Source: J Cell Biol. 2022 Mar 10;221(4):e202010065. doi: 10.1083/jcb.202010065 (PMC8919618; doi:10.1083/jcb.202010065)

Figure 1d Source Data
Blot 1

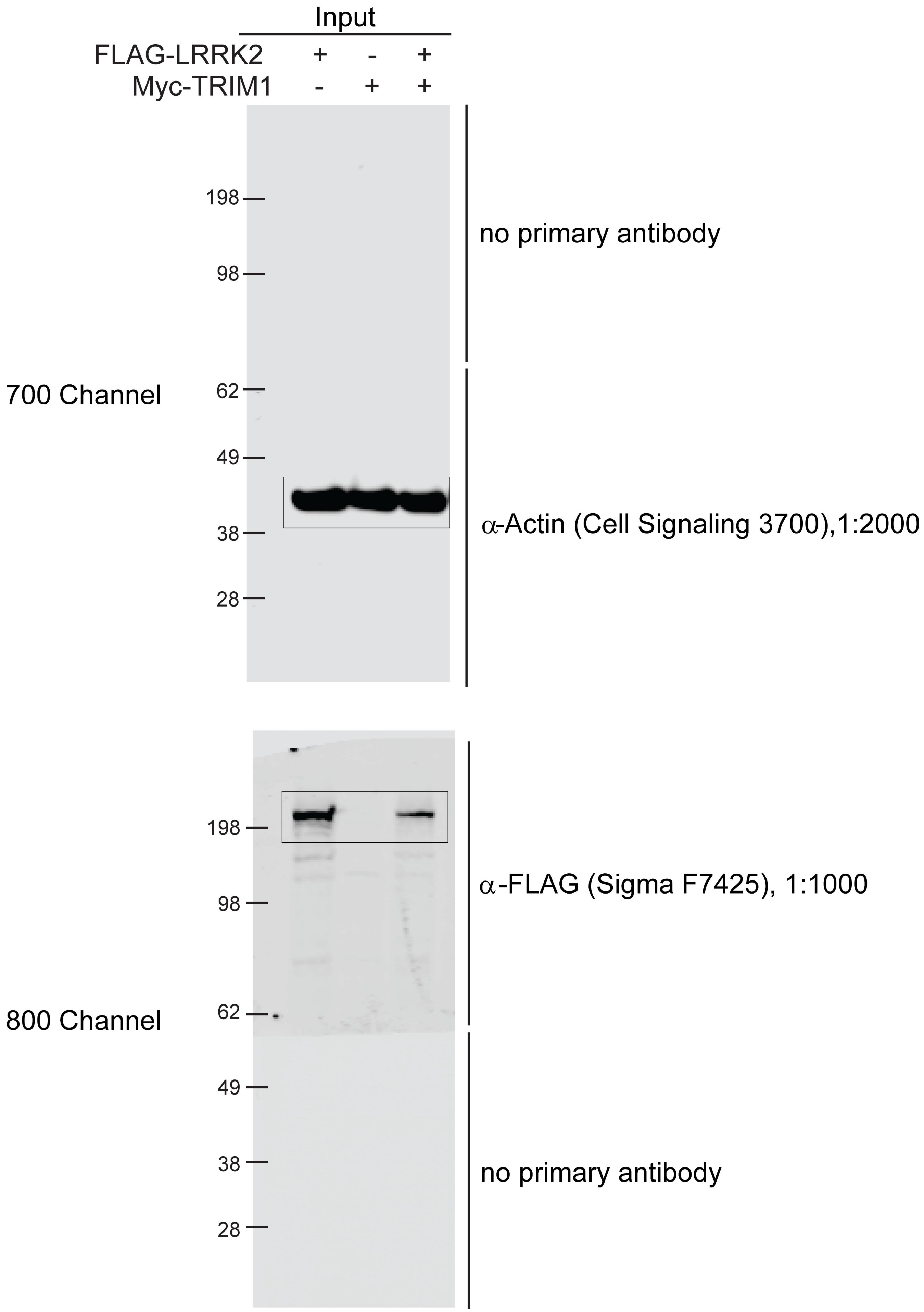

Figure 1d Source Data
Blot 2

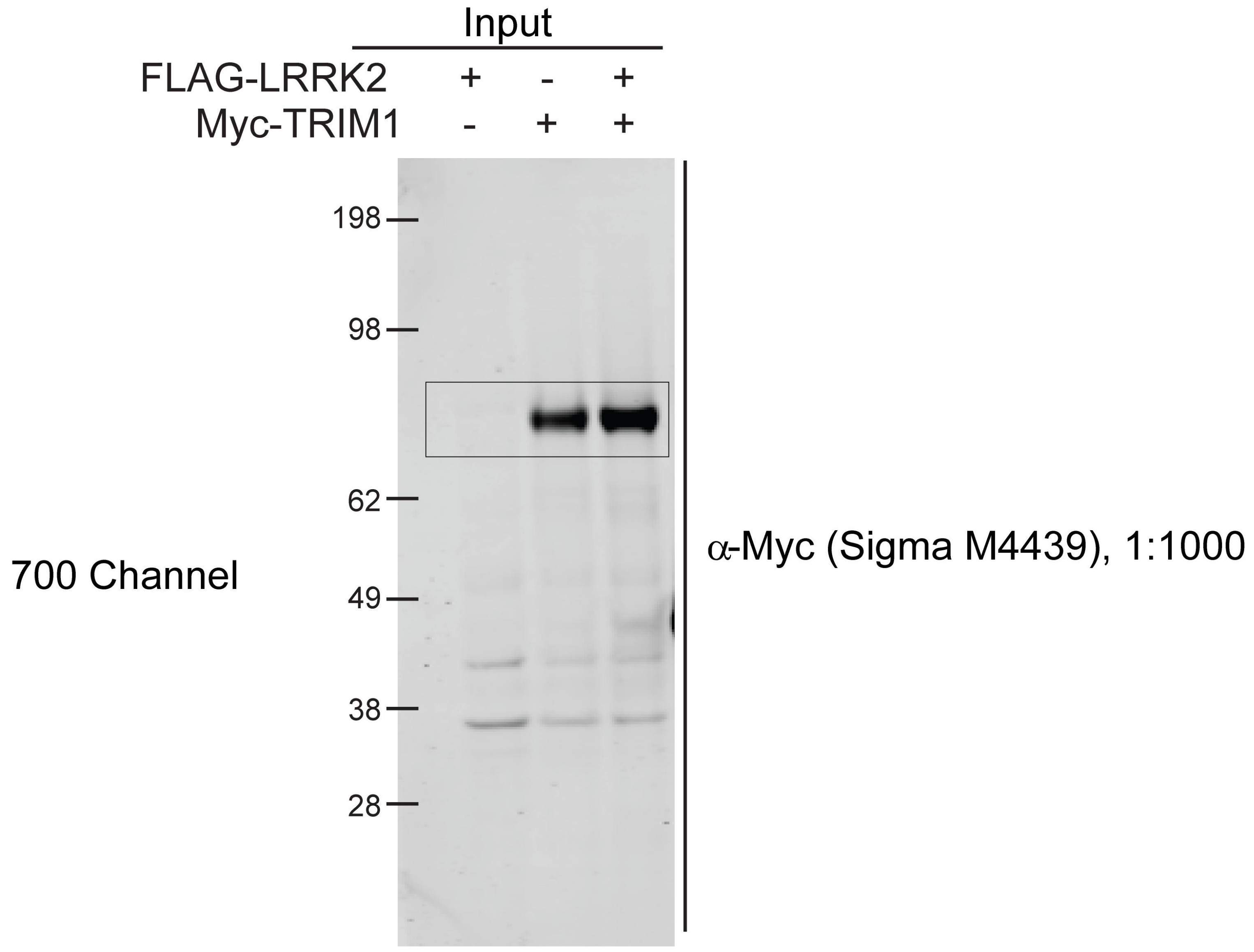

Figure 1d Source Data
Blot 3

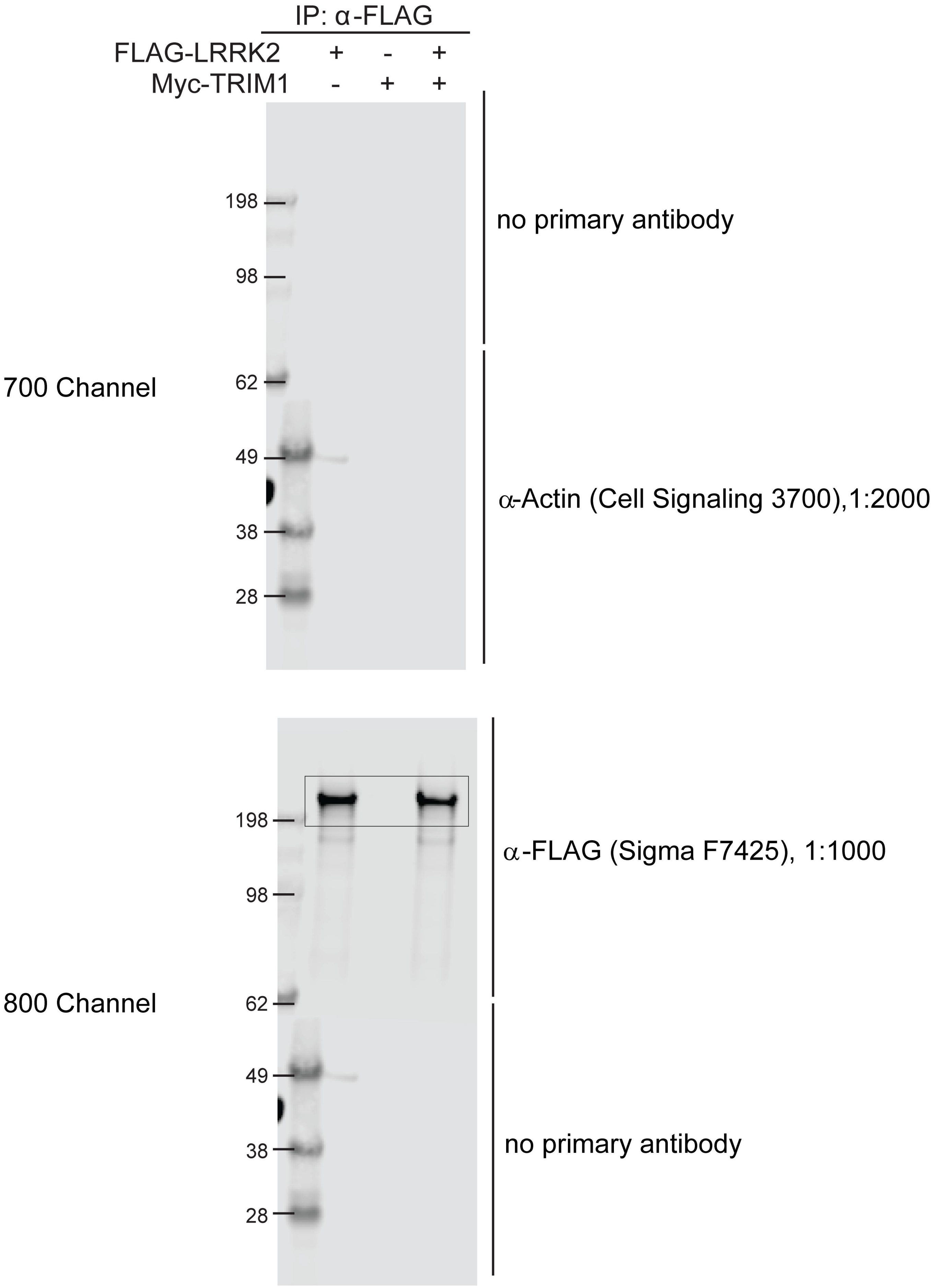

Figure 1d Source Data
Blot 4

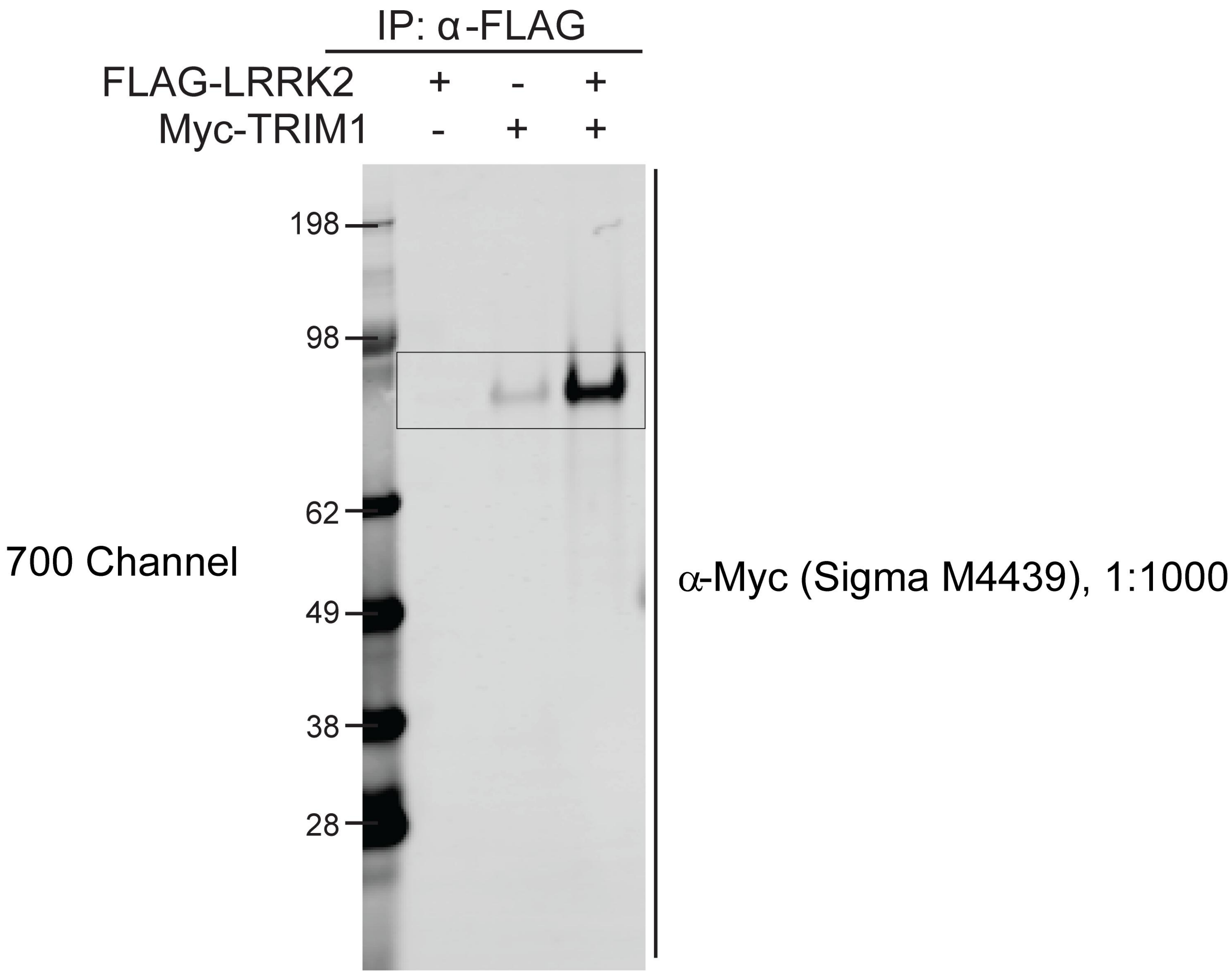

Figure 1e

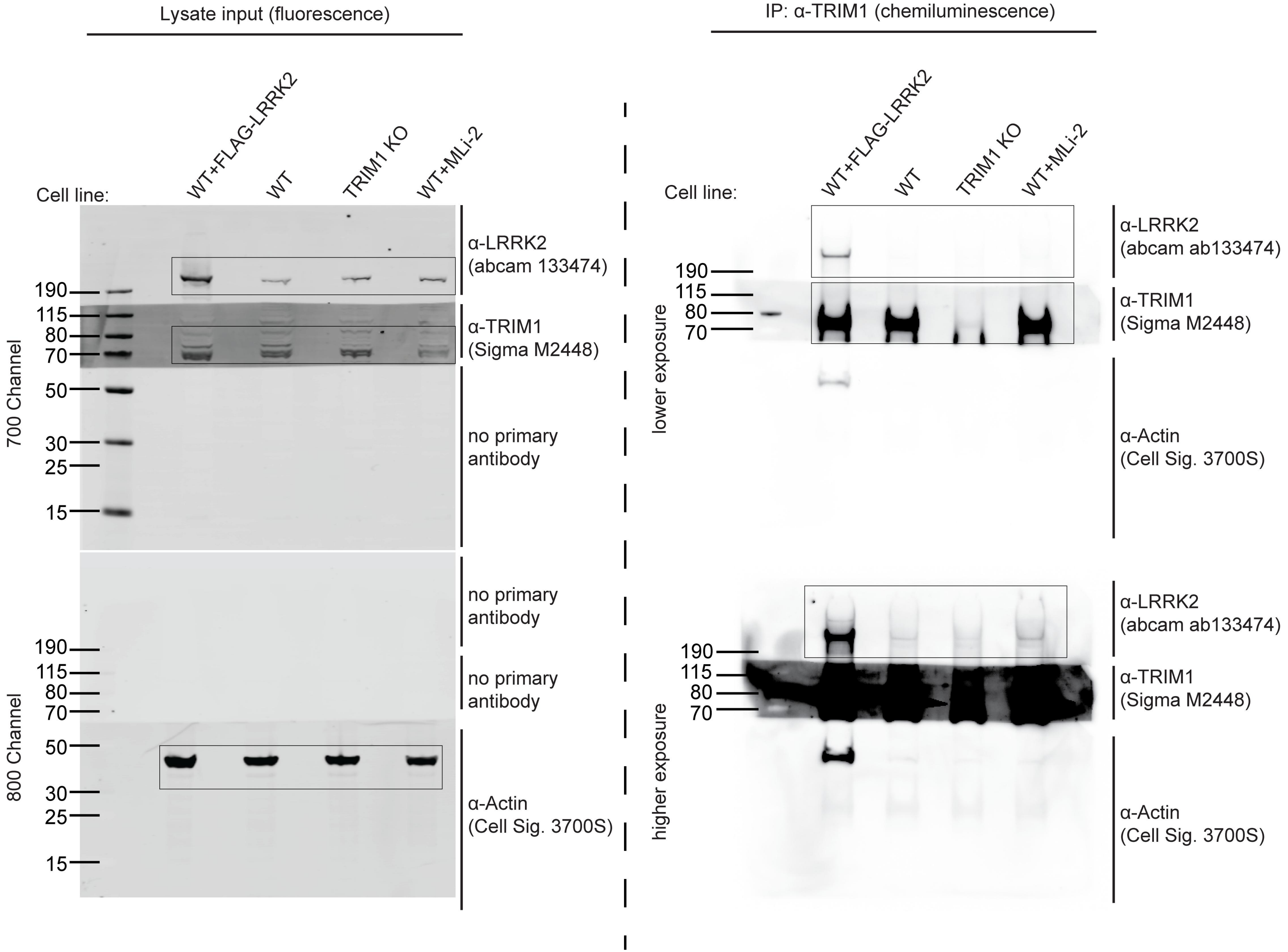

Lysate input (fluorescence)
IP: α-TRIM1 (chemiluminescence)
Cell line:
WT+FLAG-LRRK2
WT
TRIM1 KO
WT+MLi-2
α-LRRK2
(abcam 133474)
α-TRIM1
(Sigma M2448)
no primary
antibody
α-Actin
(Cell Sig. 3700S)
700 Channel
800 Channel
190
115
80
70
50
30
25
15
lower exposure
higher exposure
α-LRRK2
(abcam ab133474)

Figure 1f Source Data

Blot 1

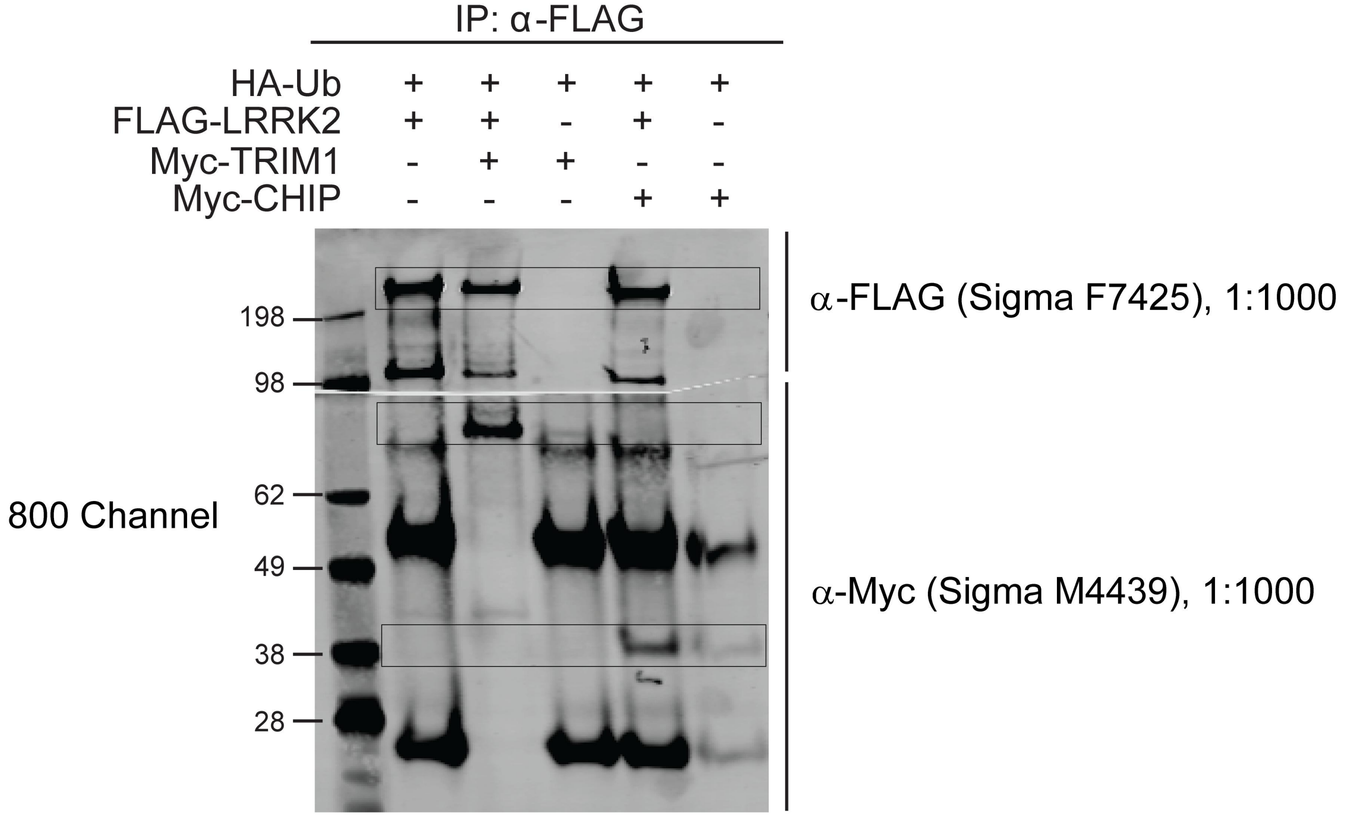

Blot 2

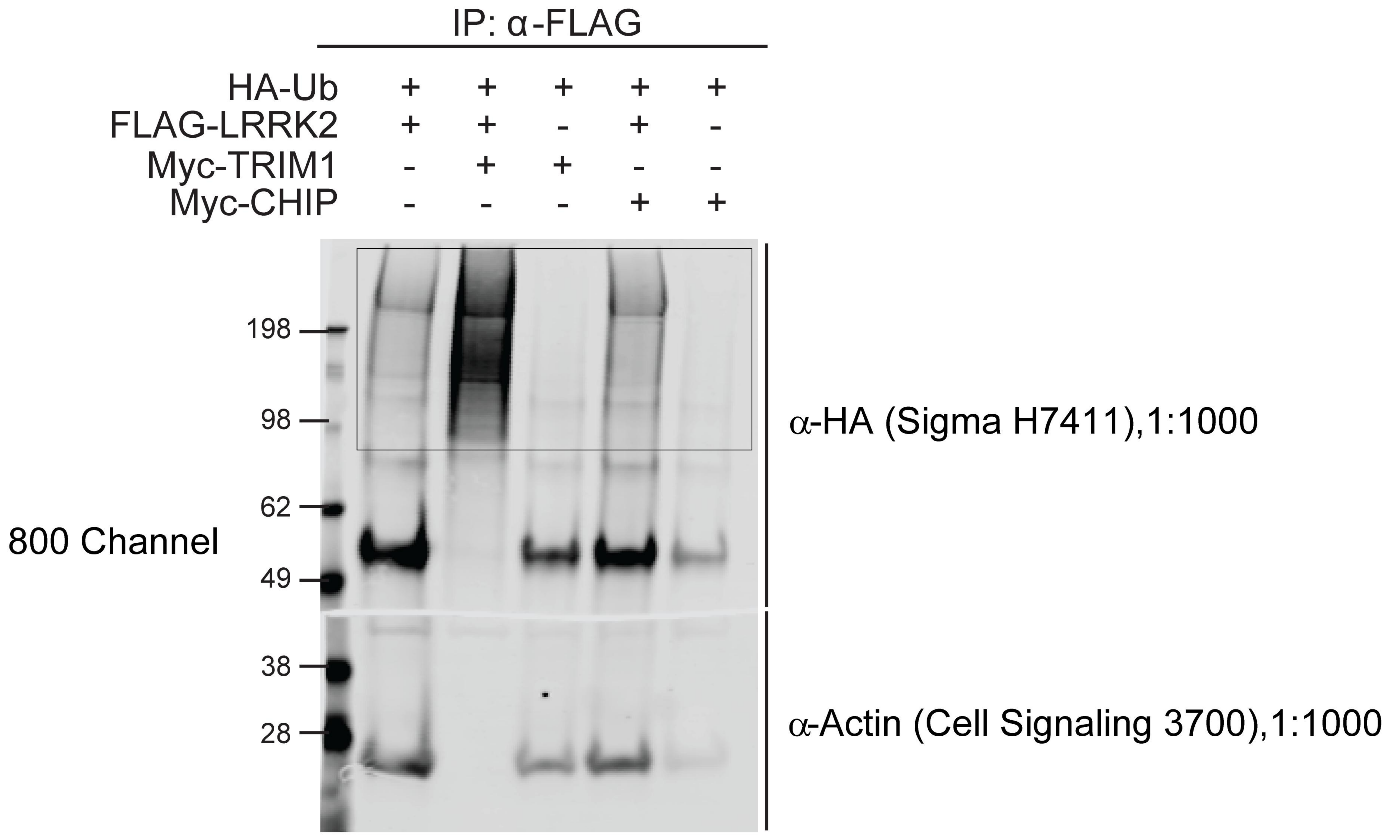

Figure 1f Source Data

Blot 3

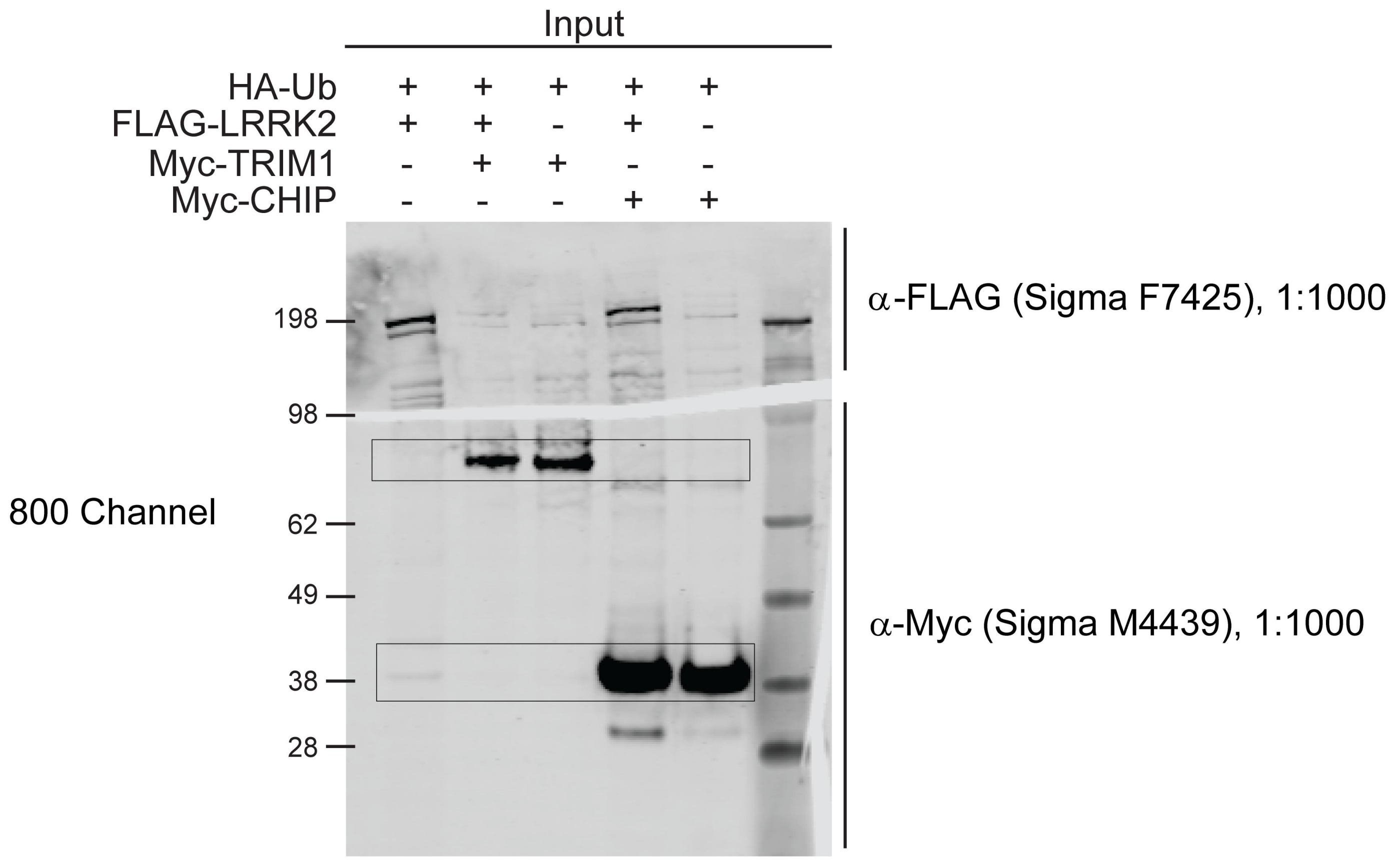

Blot 4

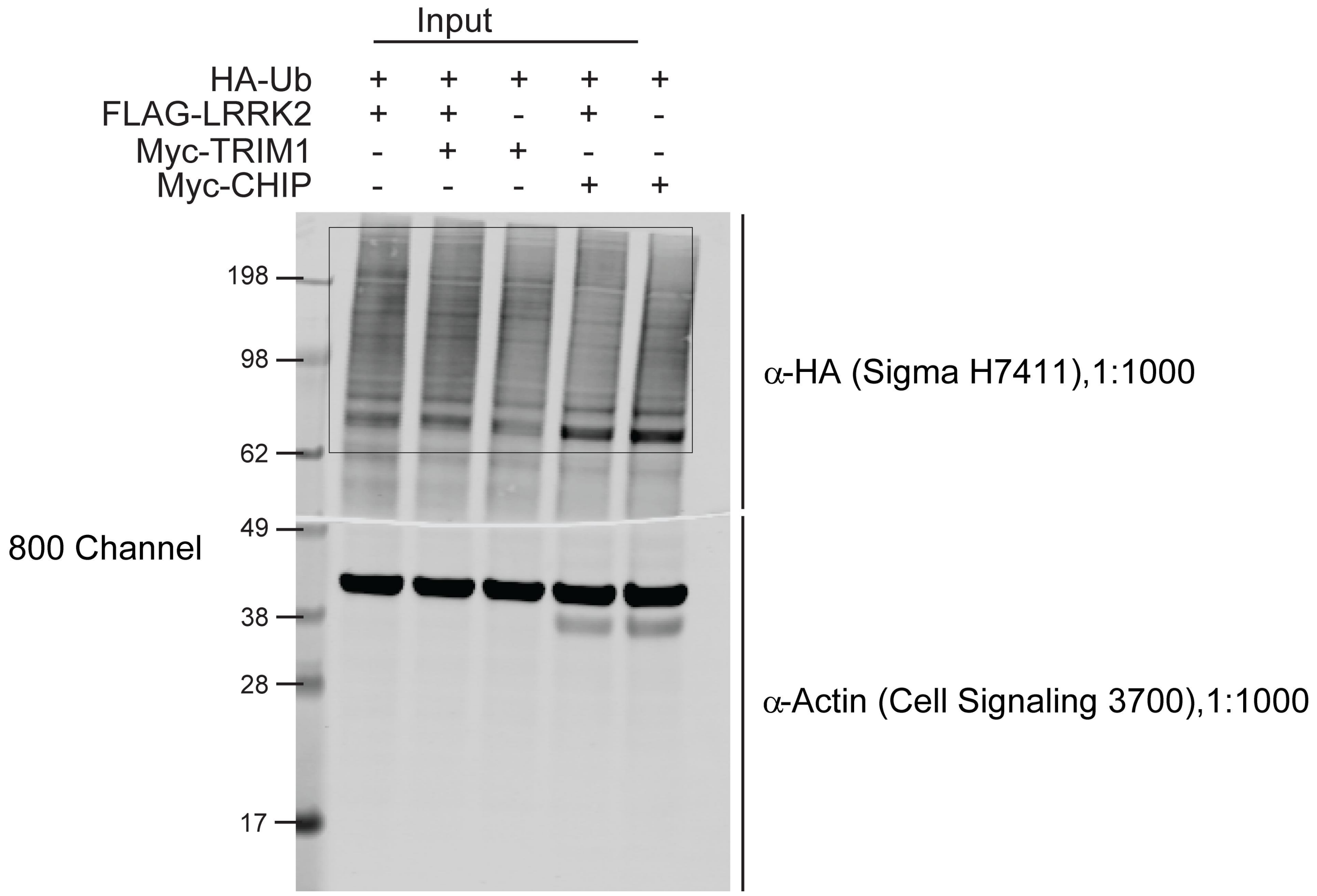

Figure 1f Source Data

Blot 5

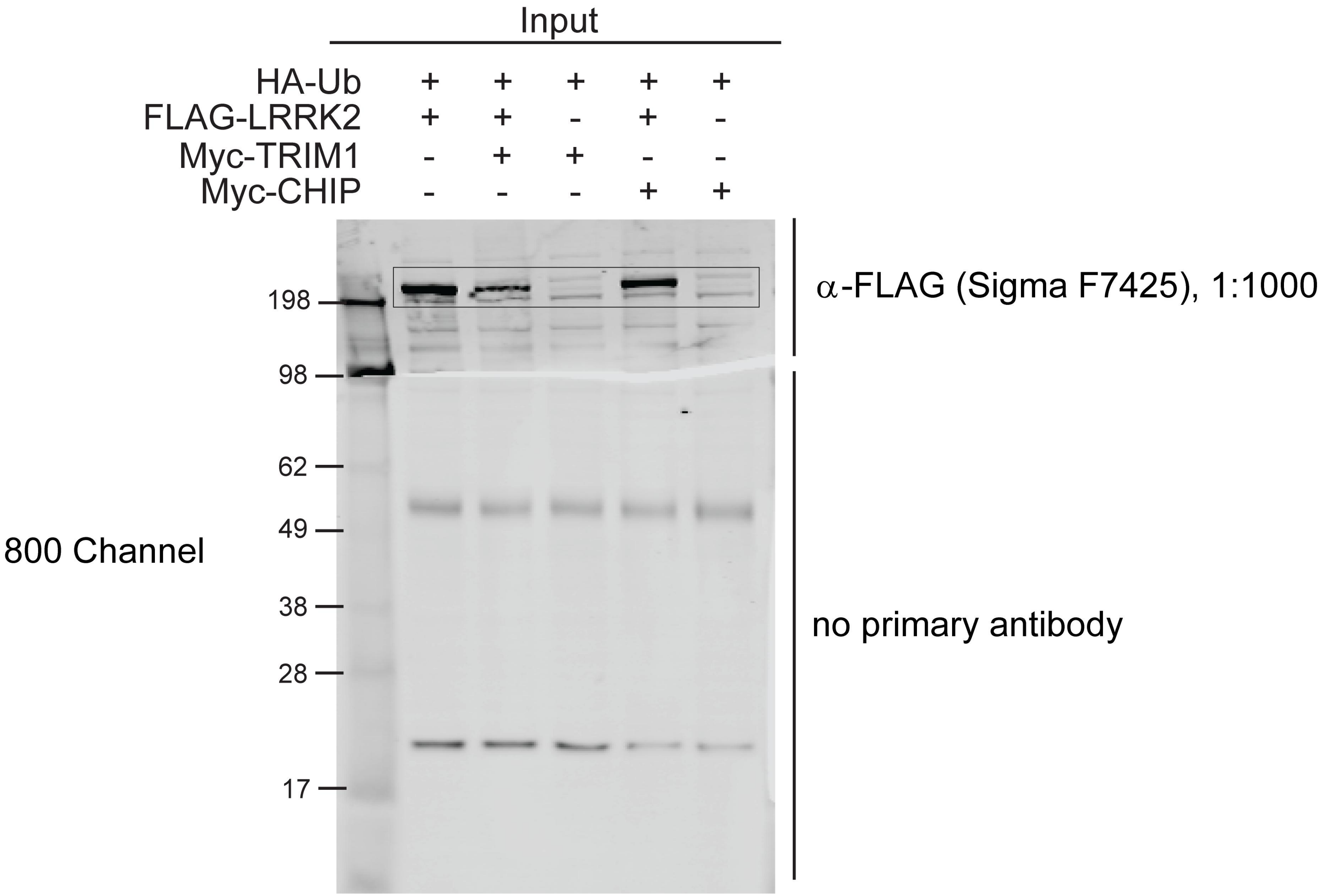

Supplement: SourceData F1 — is the source file for Fig. 1. [file JCB_202010065_SourceDataF1.pdf]
