# Supplementary material for: The E3 ligase TRIM1 ubiquitinates LRRK2 and controls its localization, degradation, and toxicity
Source: J Cell Biol. 2022 Mar 10;221(4):e202010065. doi: 10.1083/jcb.202010065 (PMC8919618; doi:10.1083/jcb.202010065)

# Figure 3a

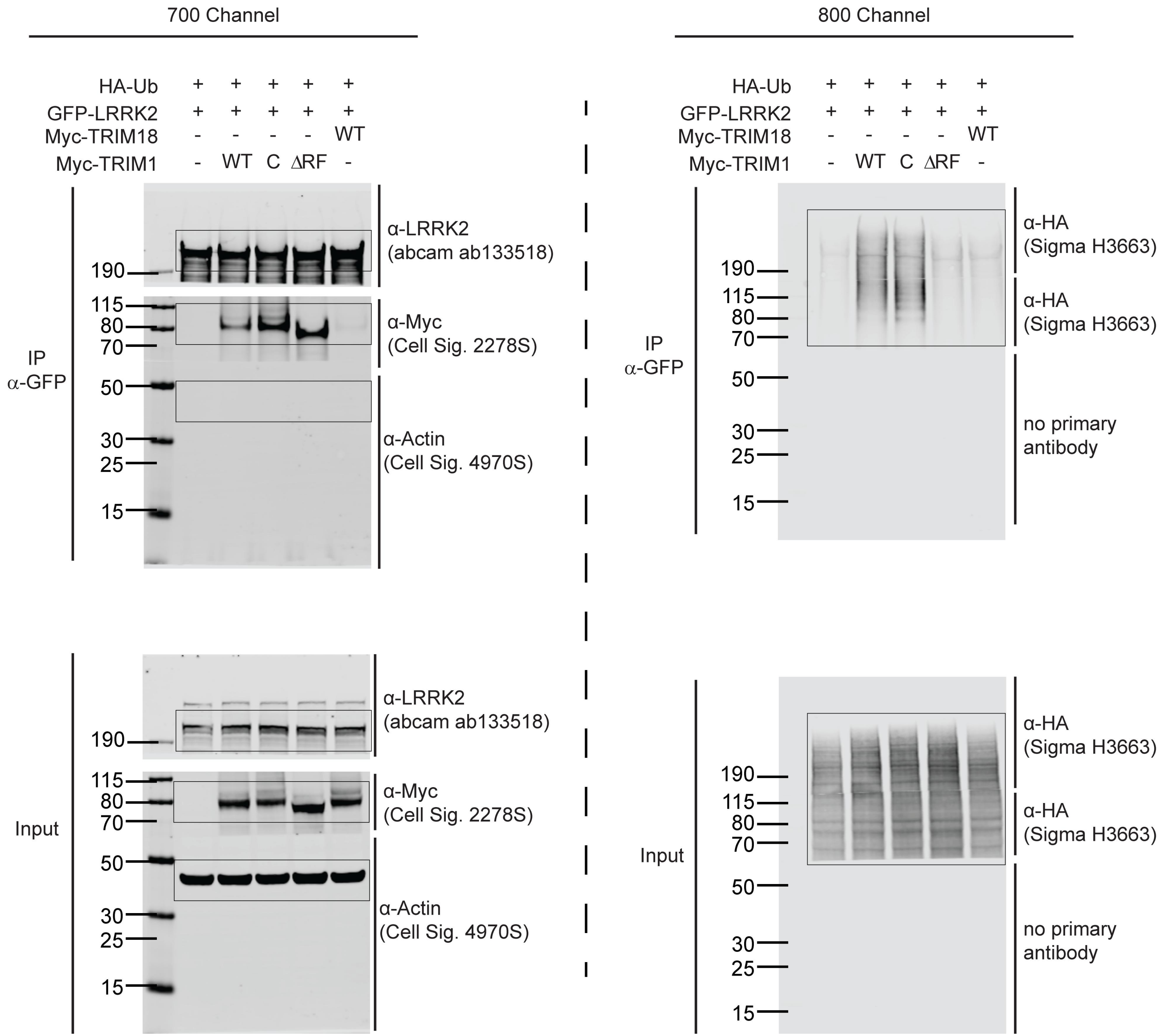
700 Channel
800 Channel
HA-Ub + + + + +
GFP-LRRK2 + + + + +
Myc-TRIM18 - - - - WT
Myc-TRIM1 - WT C ΔRF -
IP α-GFP
α-LRRK2 (abcam ab133518)
α-Myc (Cell Sig. 2278S)
α-Actin (Cell Sig. 4970S)
α-HA (Sigma H3663)
no primary antibody
Input
190
115
80
70
50
30
25
15

# Figure 3e Source Data

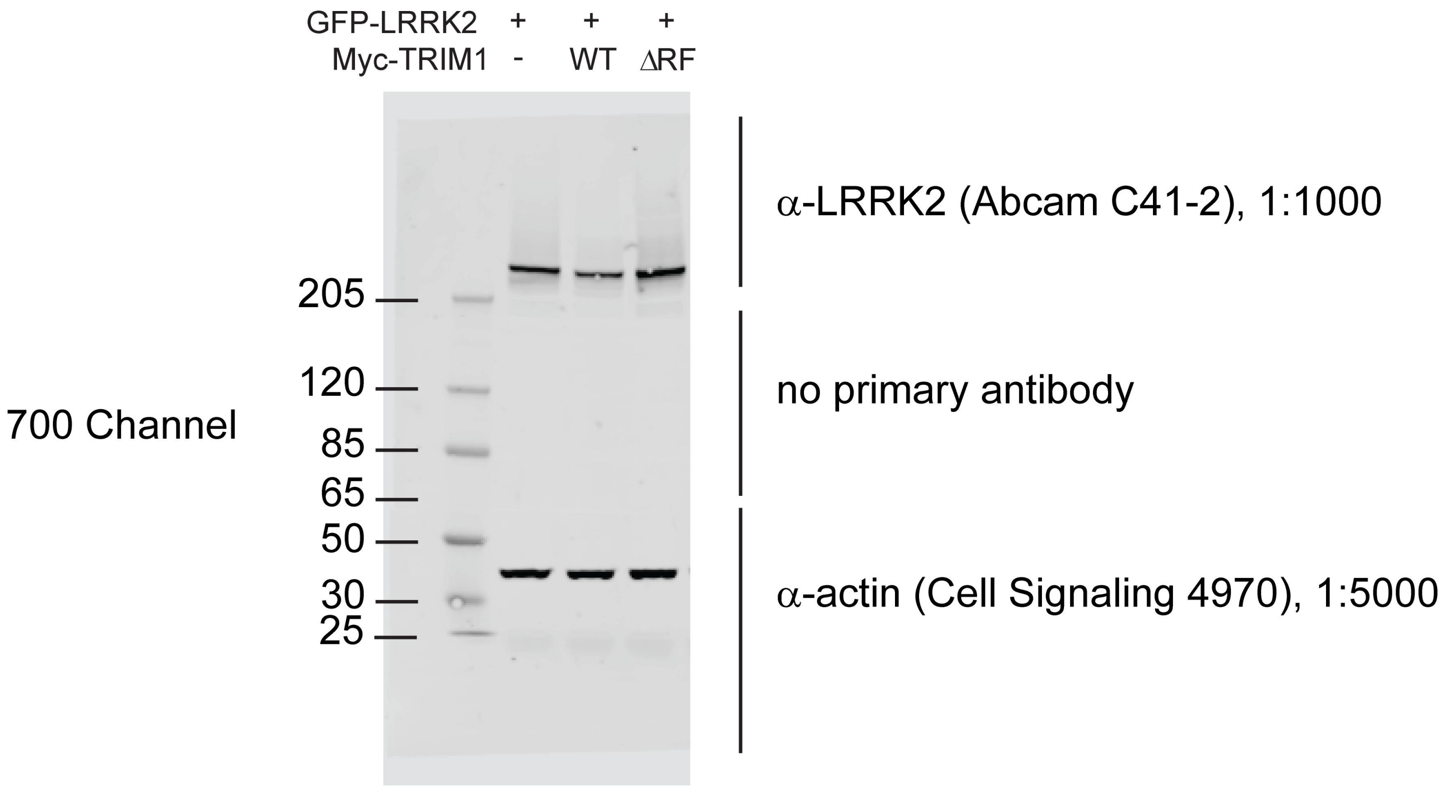


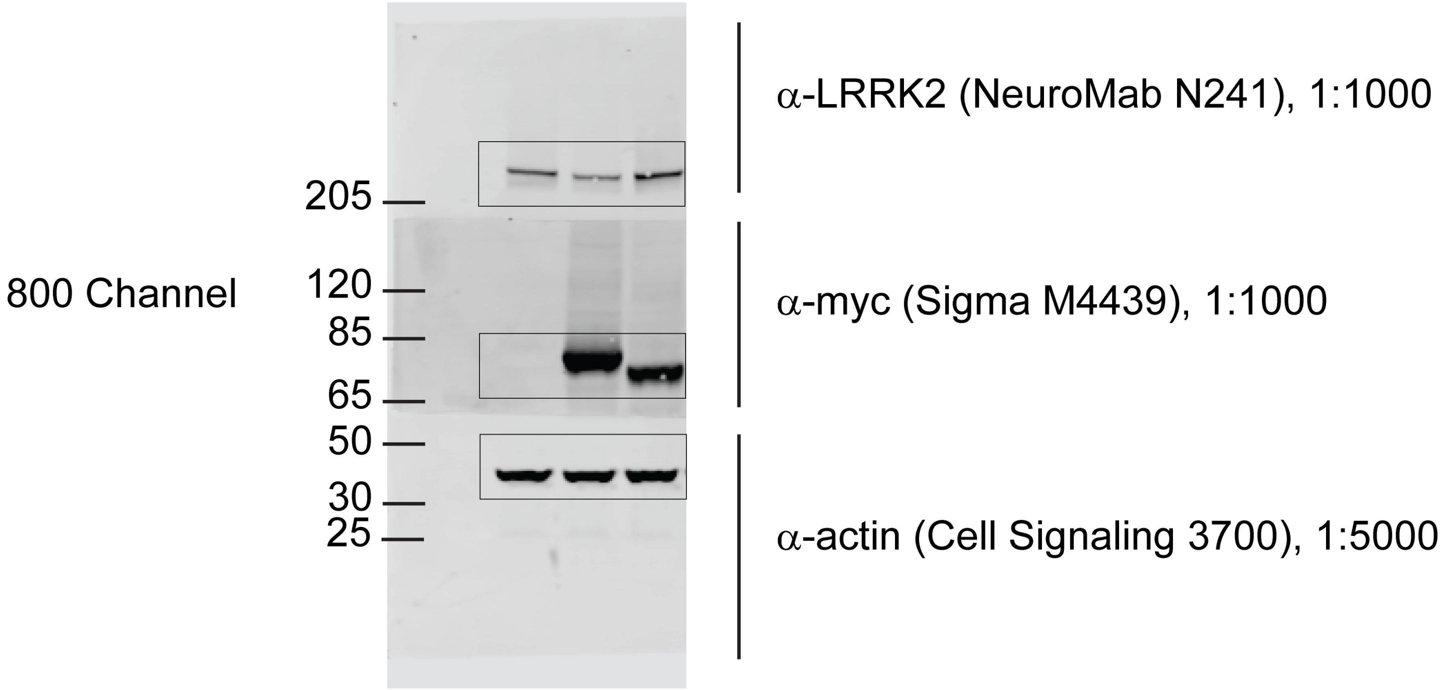

**Source Data F3h**
**Blot 1**

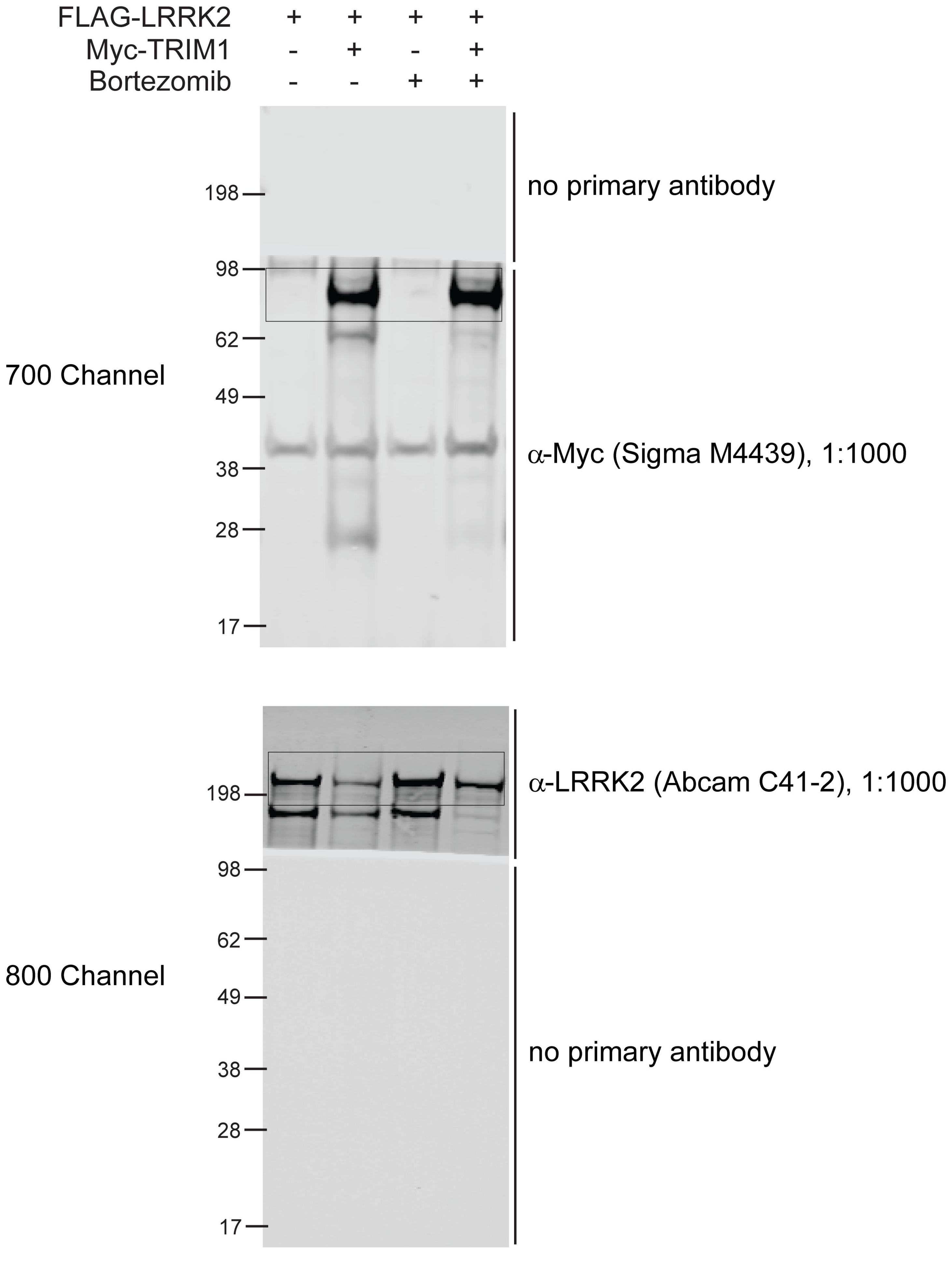

# Source Data F3h
# Blot 2

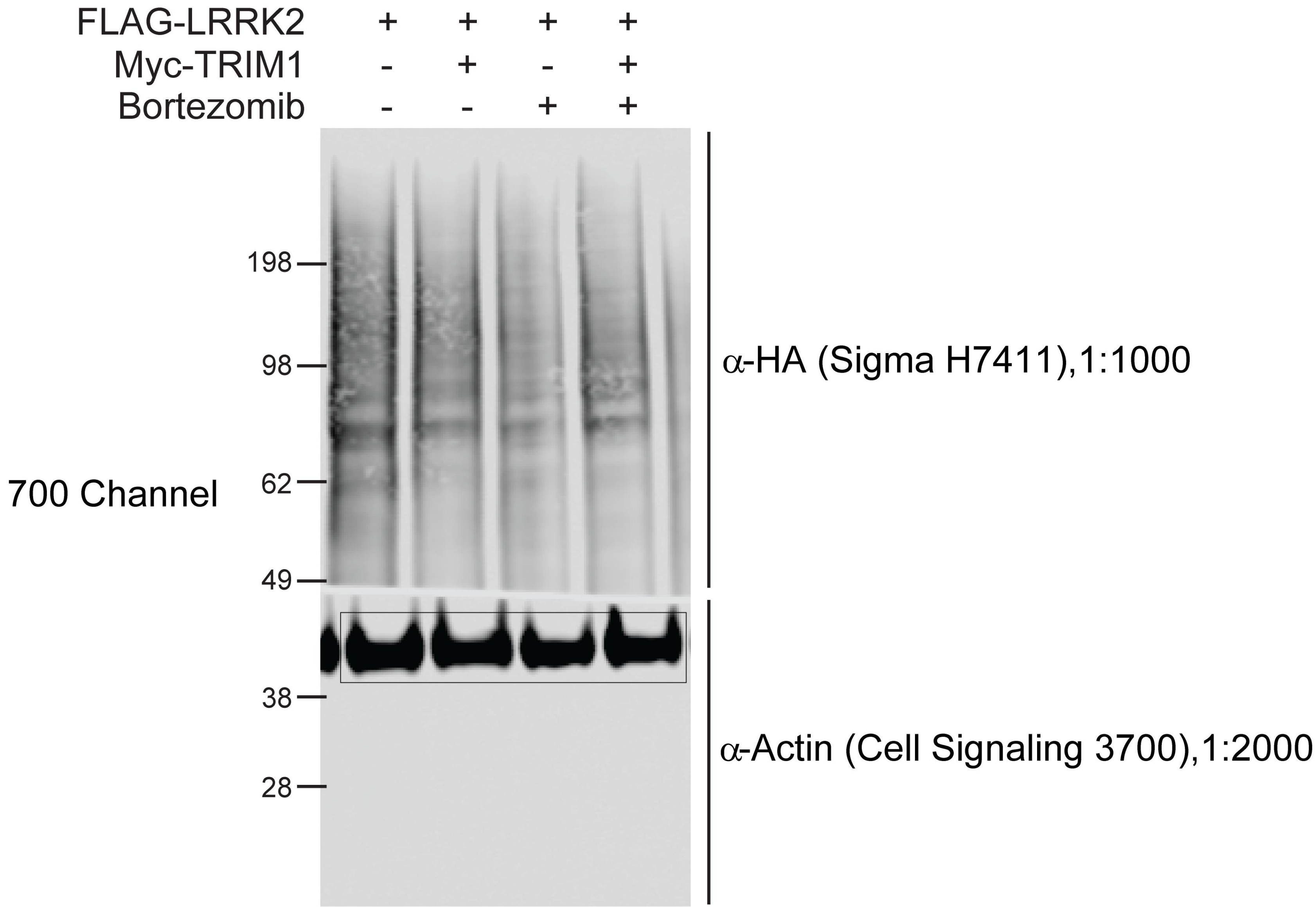

Supplement: SourceData F3 — is the source file for Fig.3. [file JCB_202010065_SourceDataF3.pdf]
