# Supplementary figures and images for: The E3 ligase TRIM1 ubiquitinates LRRK2 and controls its localization, degradation, and toxicity
Source: J Cell Biol. 2022 Mar 10;221(4):e202010065. doi: 10.1083/jcb.202010065 (PMC8919618; doi:10.1083/jcb.202010065)

**Figure 4e Source Data**

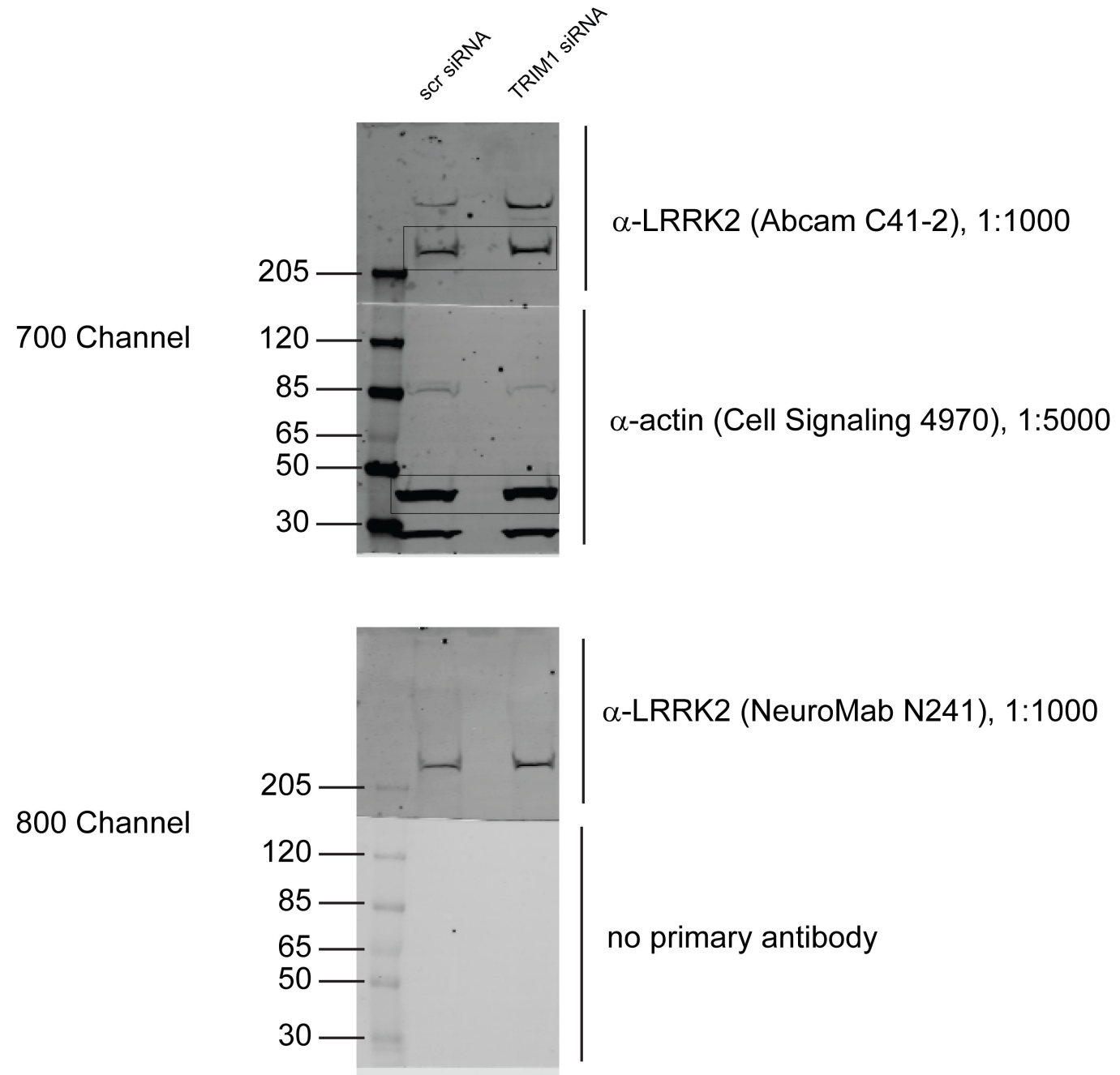

Figure 4f Source Data

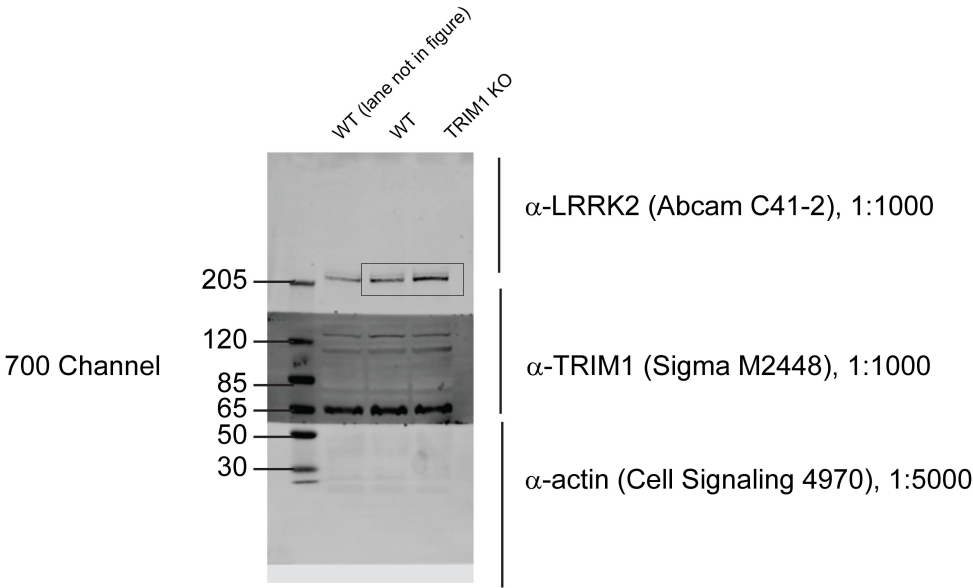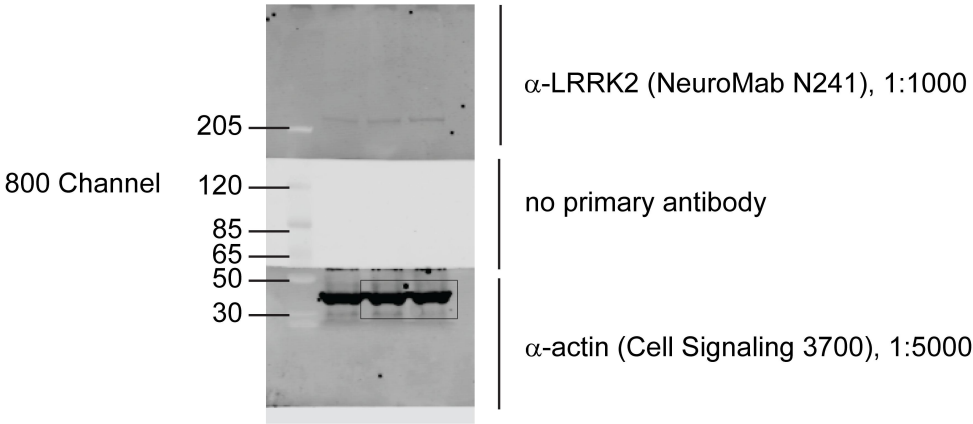

Supplement: SourceData F4 — is the source file for Fig. 4. [file JCB_202010065_SourceDataF4.pdf]

Figure 6c Source Data

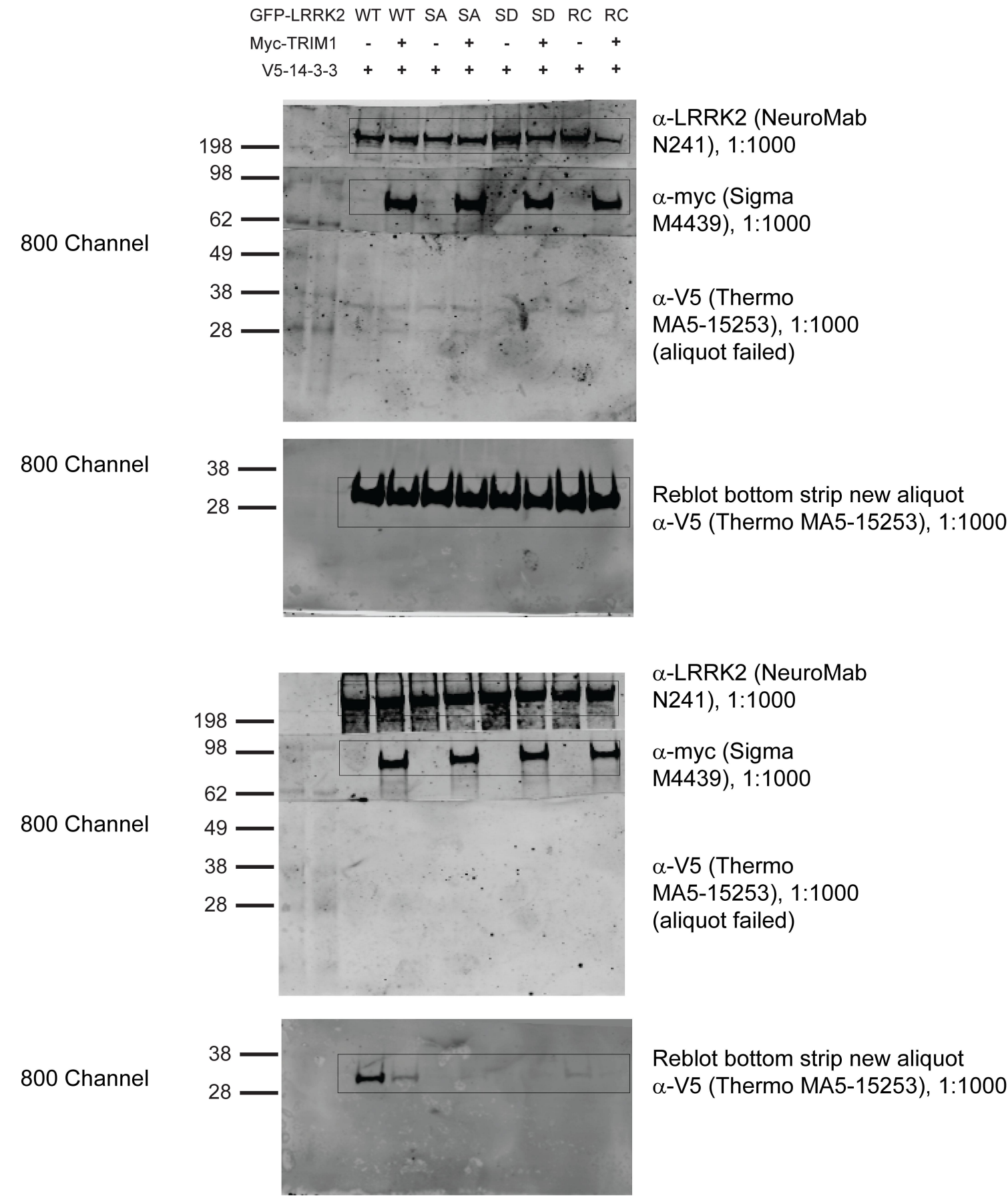

Figure 6e Source Data--Blot 1

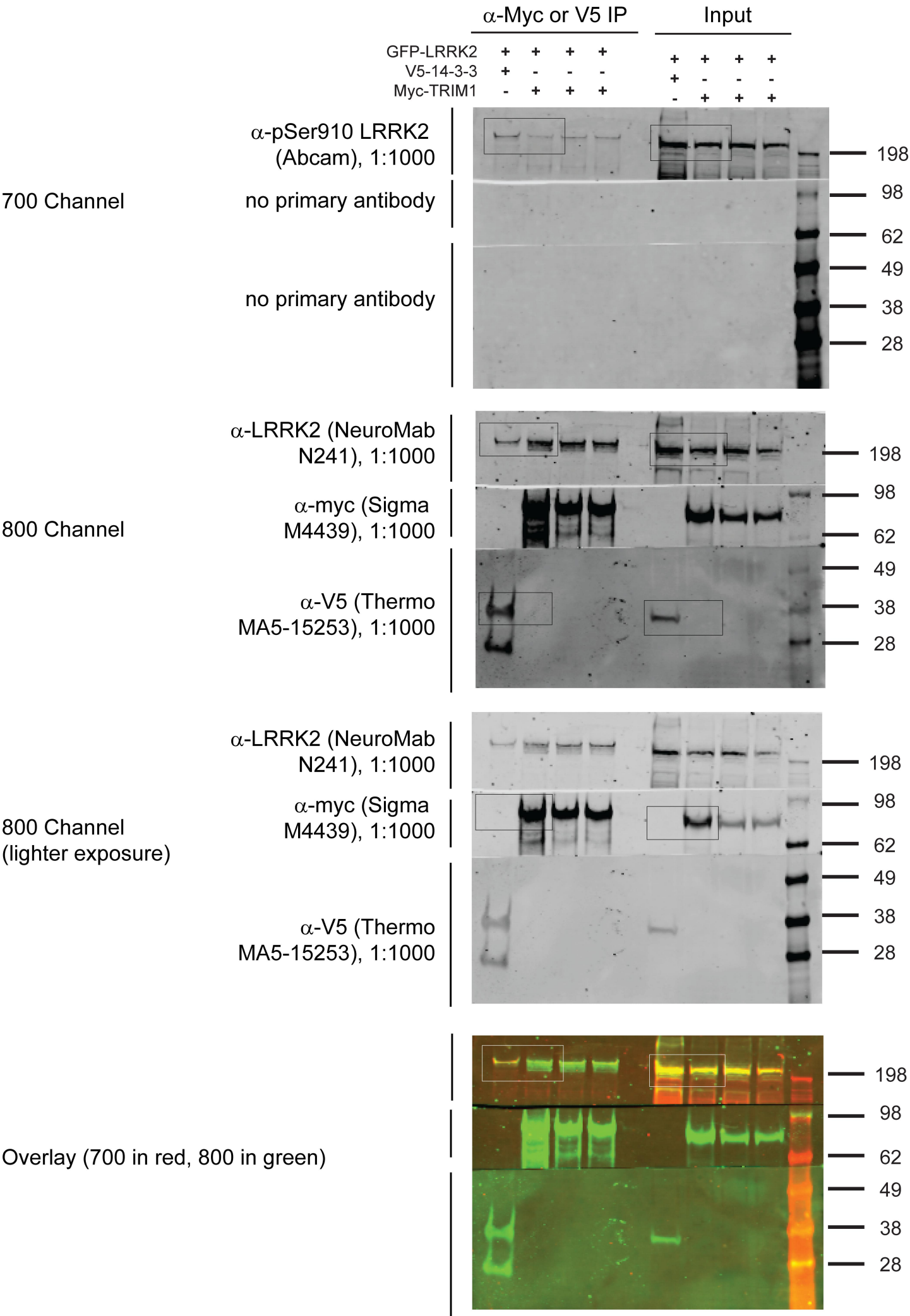

Figure 6e Source Data--blot 2

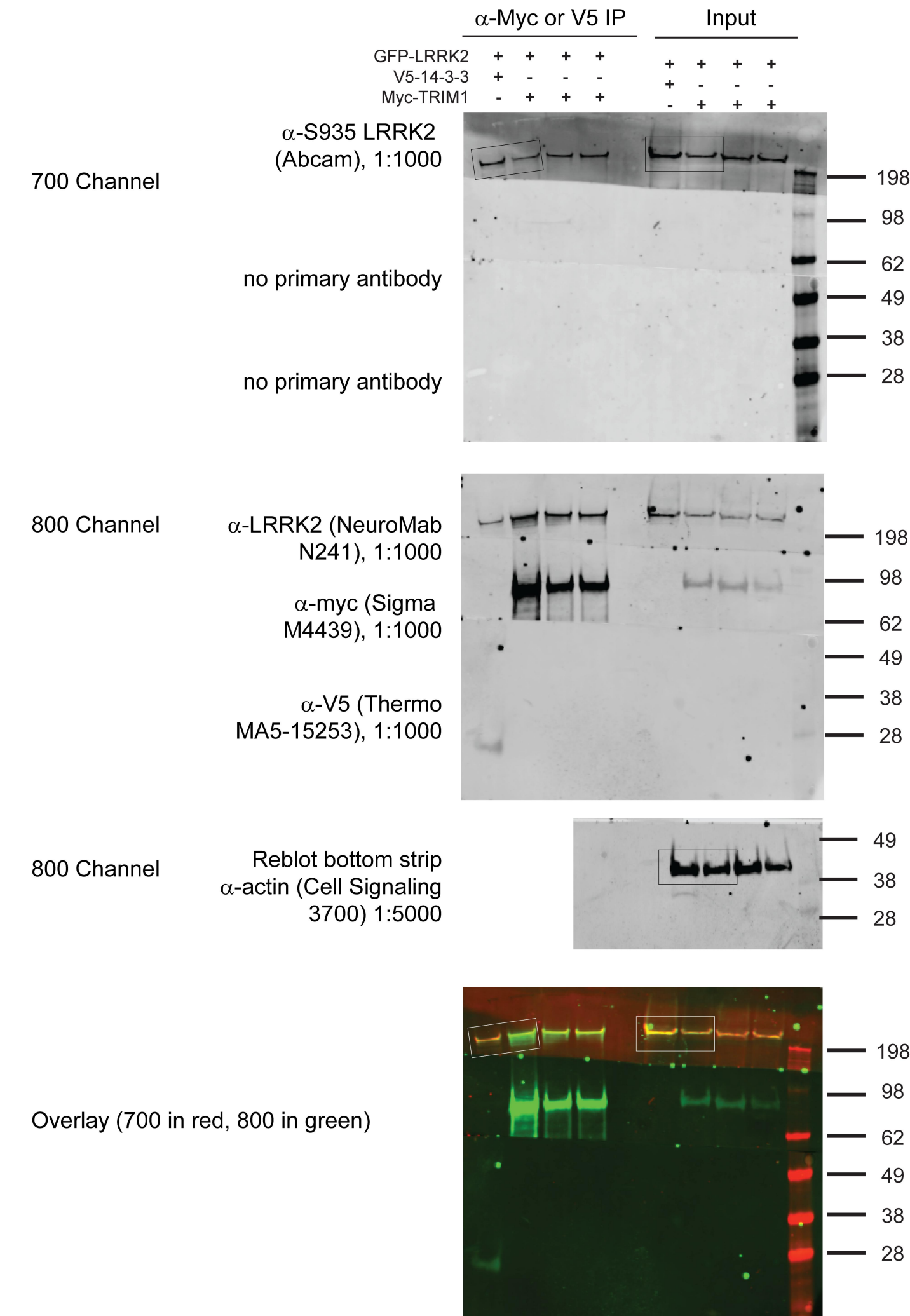

Supplement: SourceData F6 — is the source file for Fig.6. [file JCB_202010065_SourceDataF6.pdf]

### Figure 7a

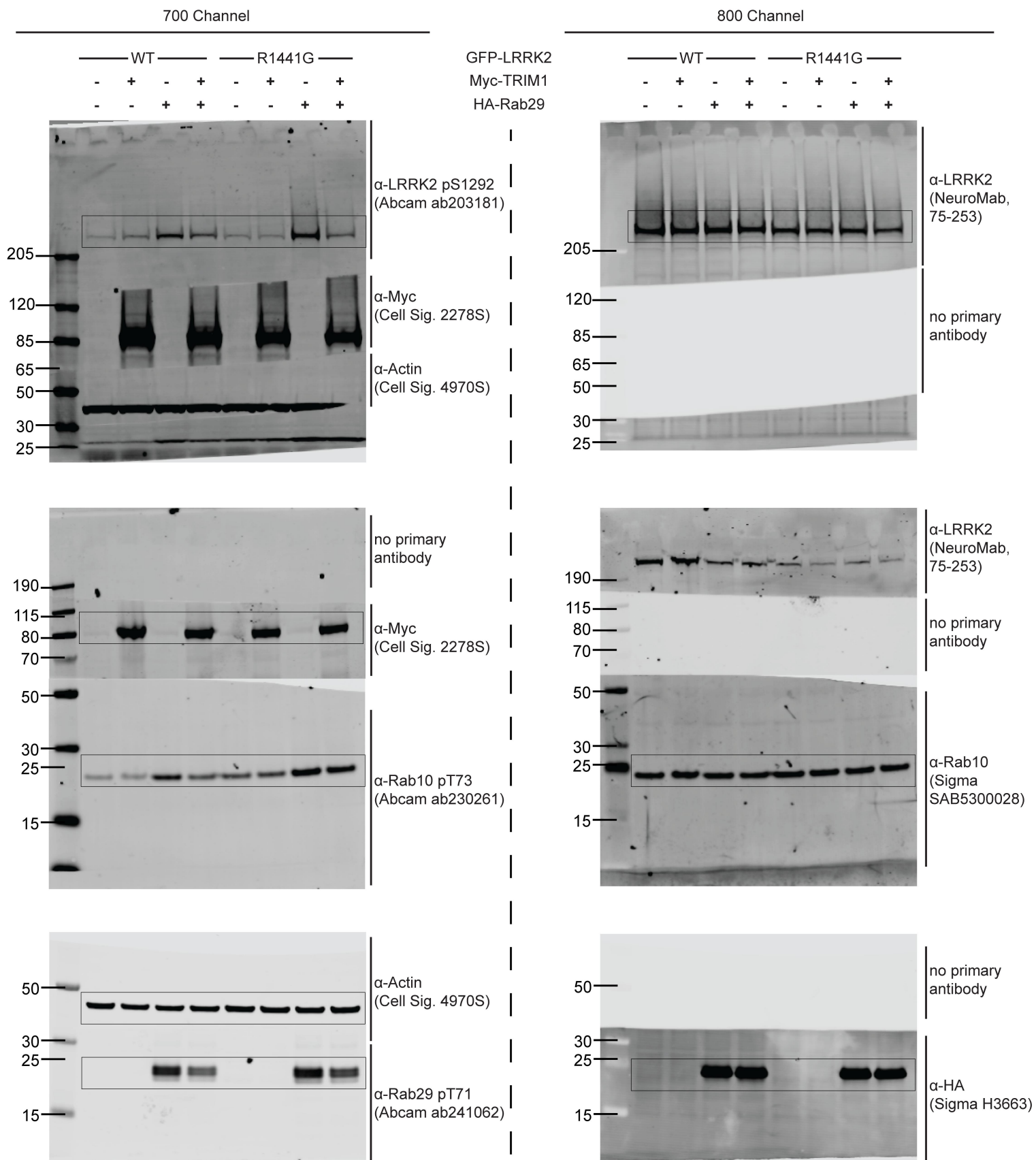

Supplement: SourceData F7 — is the source file for Fig.7. [file JCB_202010065_SourceDataF7.pdf]

Figure 8a\_page1

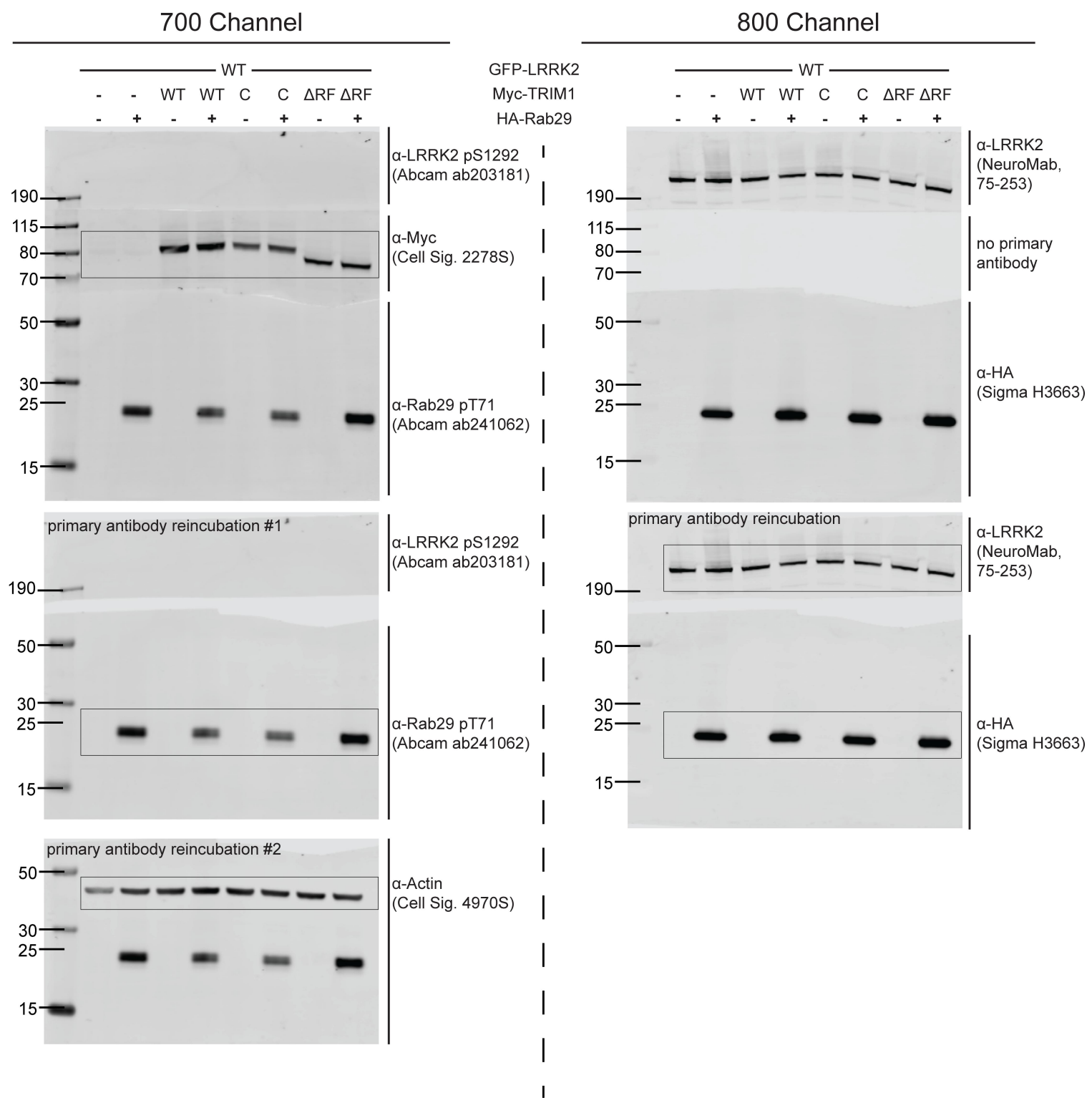

## Figure 8a\_page2

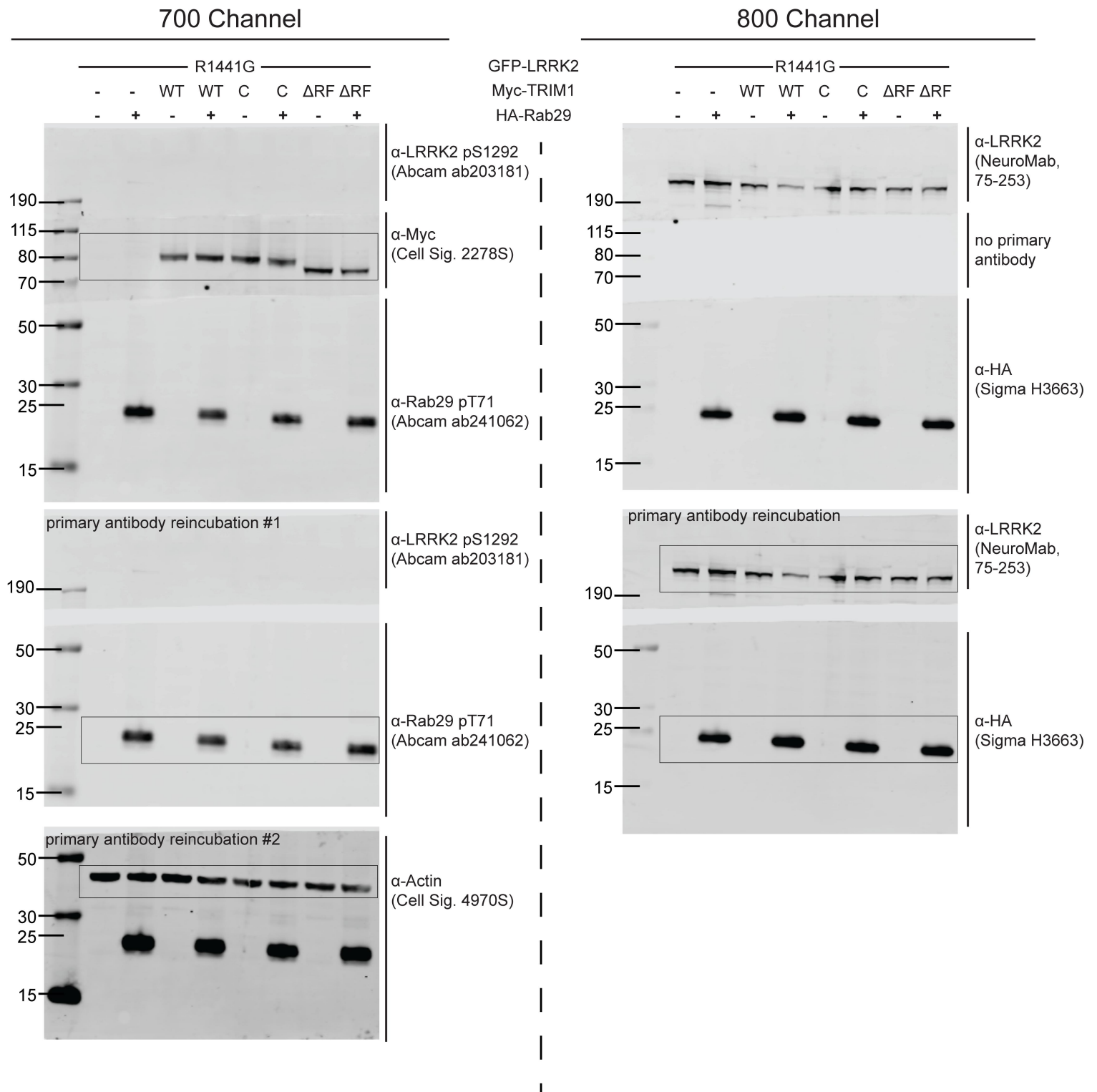

Figure 8b\_page1

700 Channel

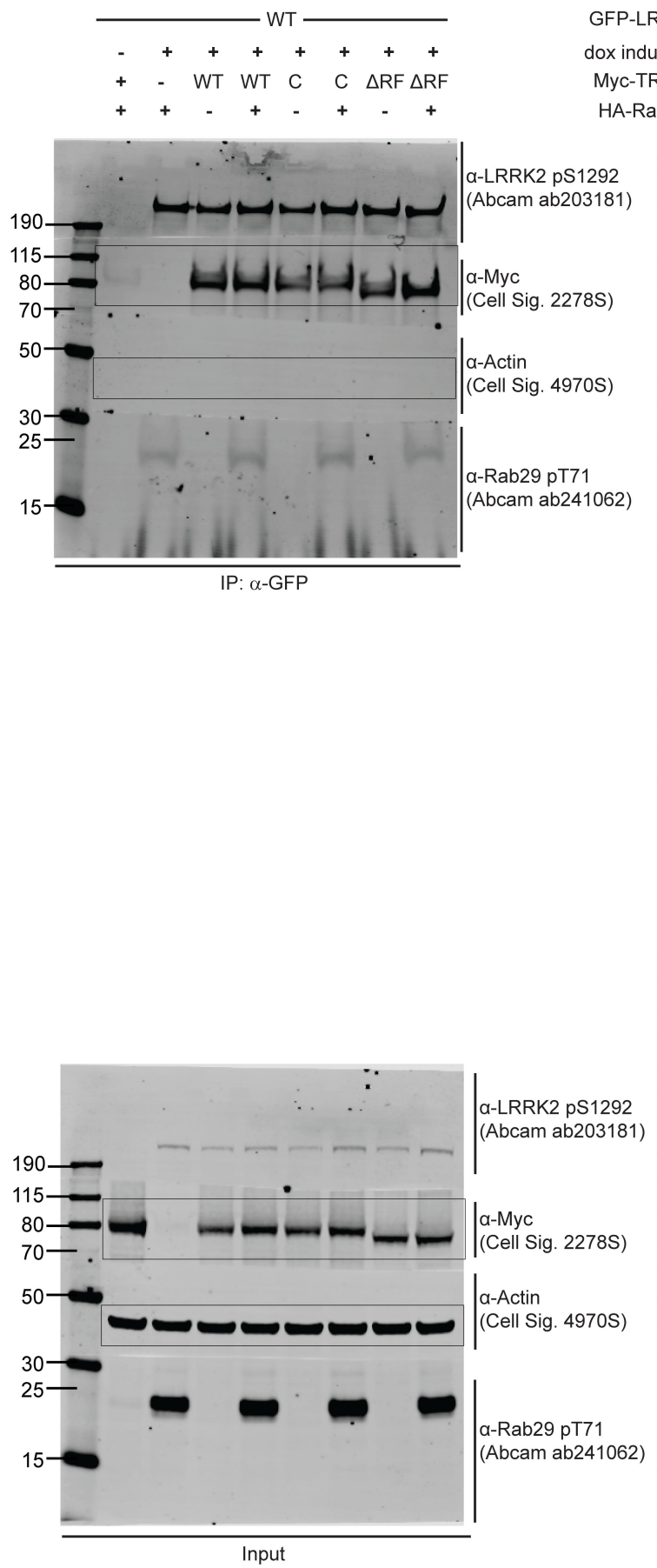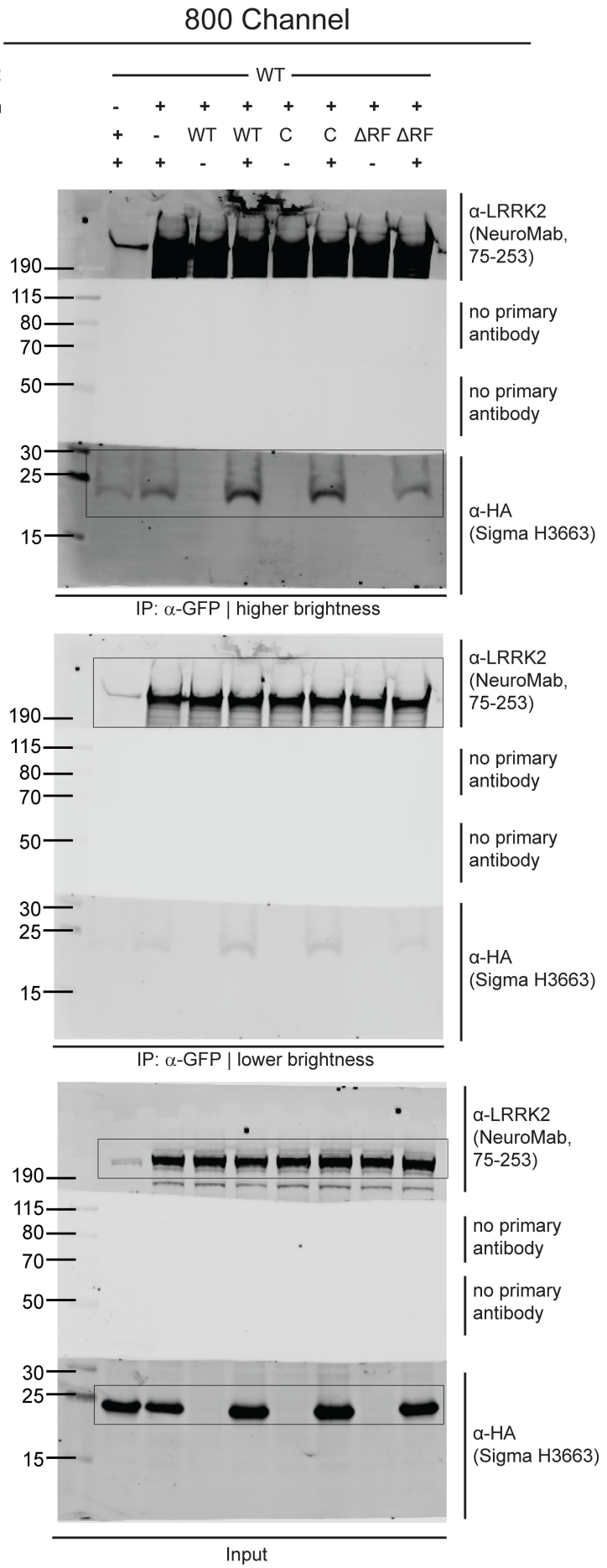

Figure 8b\_page2

700 Channel

800 Channel

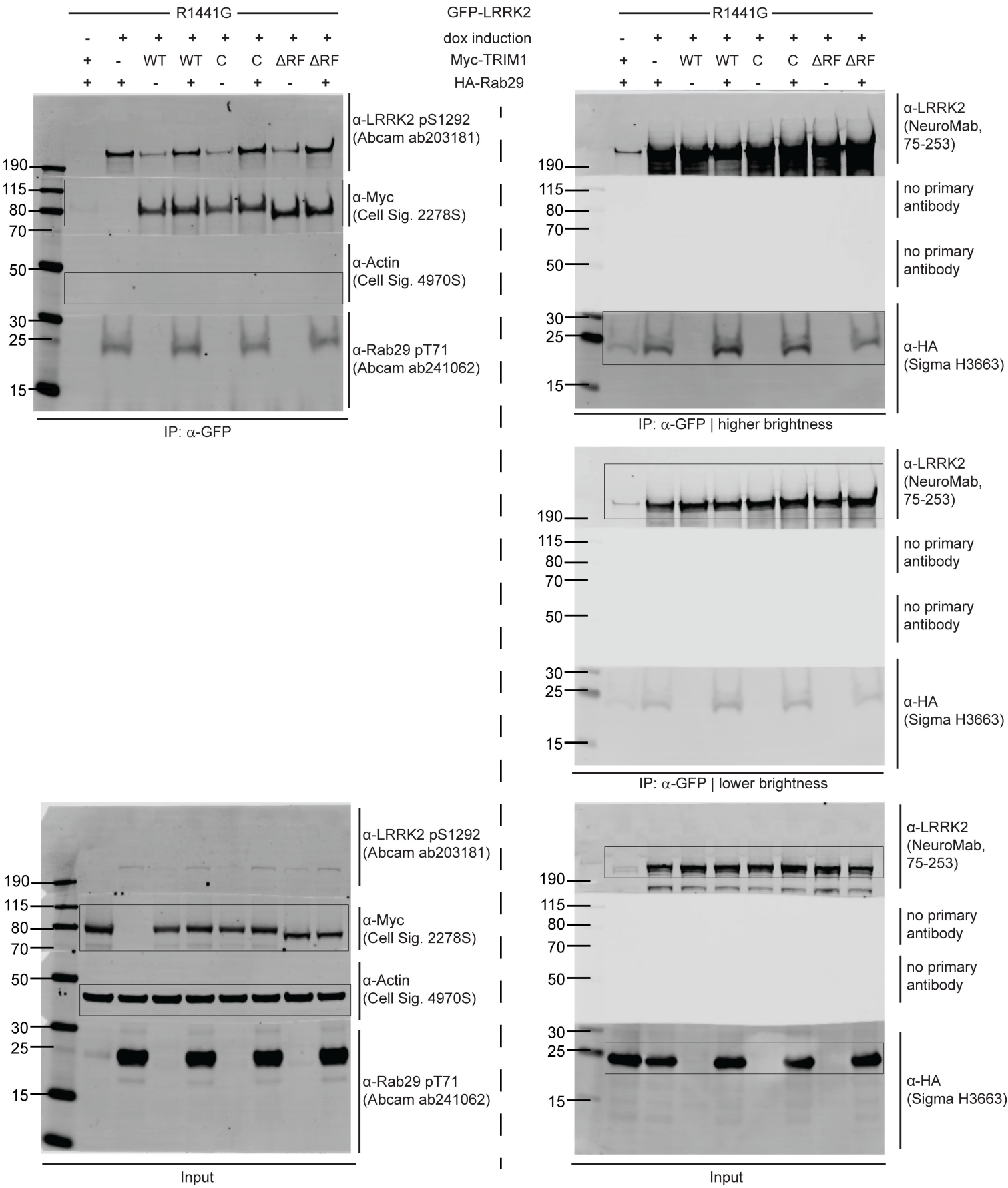

Supplement: SourceData F8 — is the source file for Fig. 8. [file JCB_202010065_SourceDataF8.pdf]

## Source Data FS2a

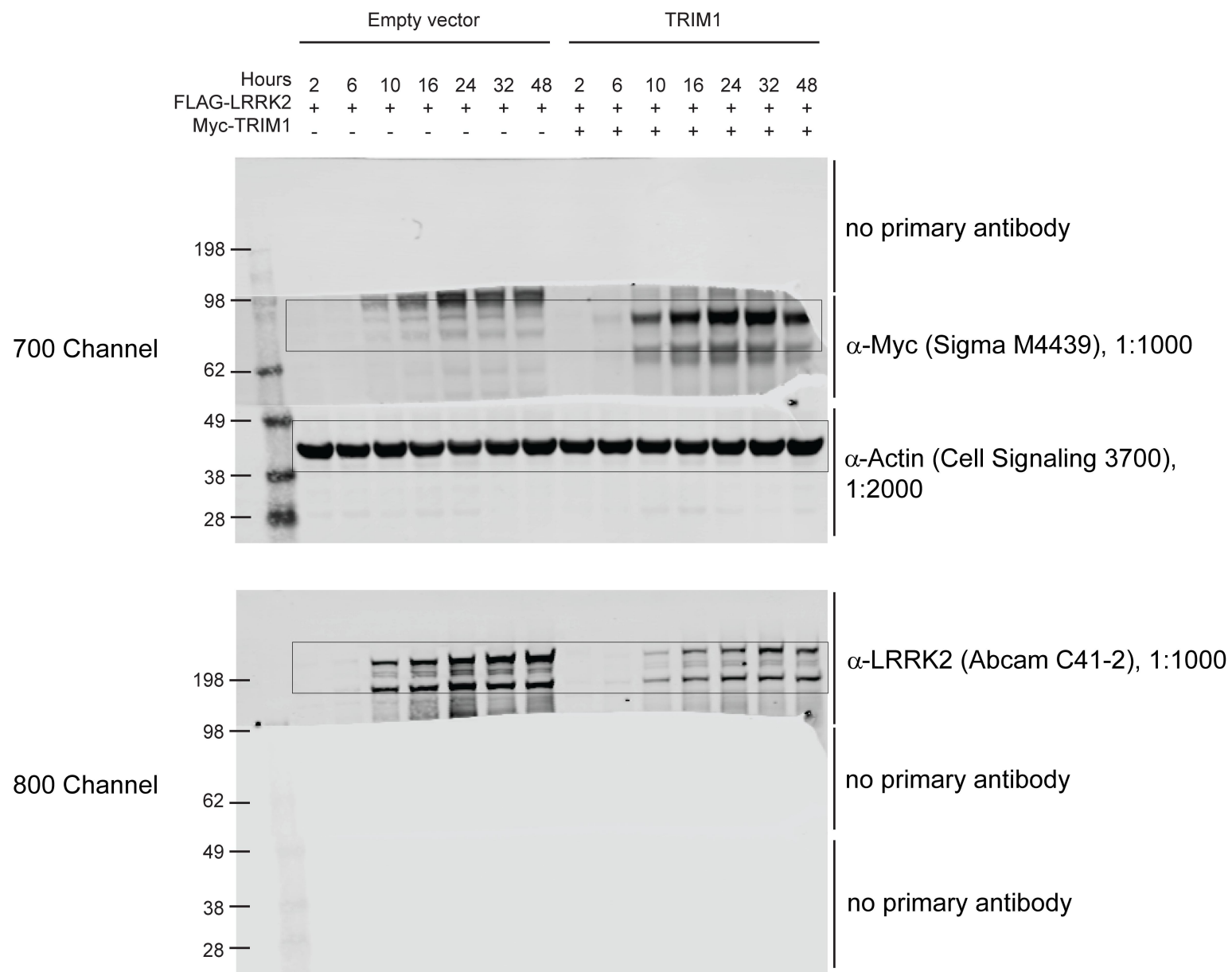

## Source Data FS2c

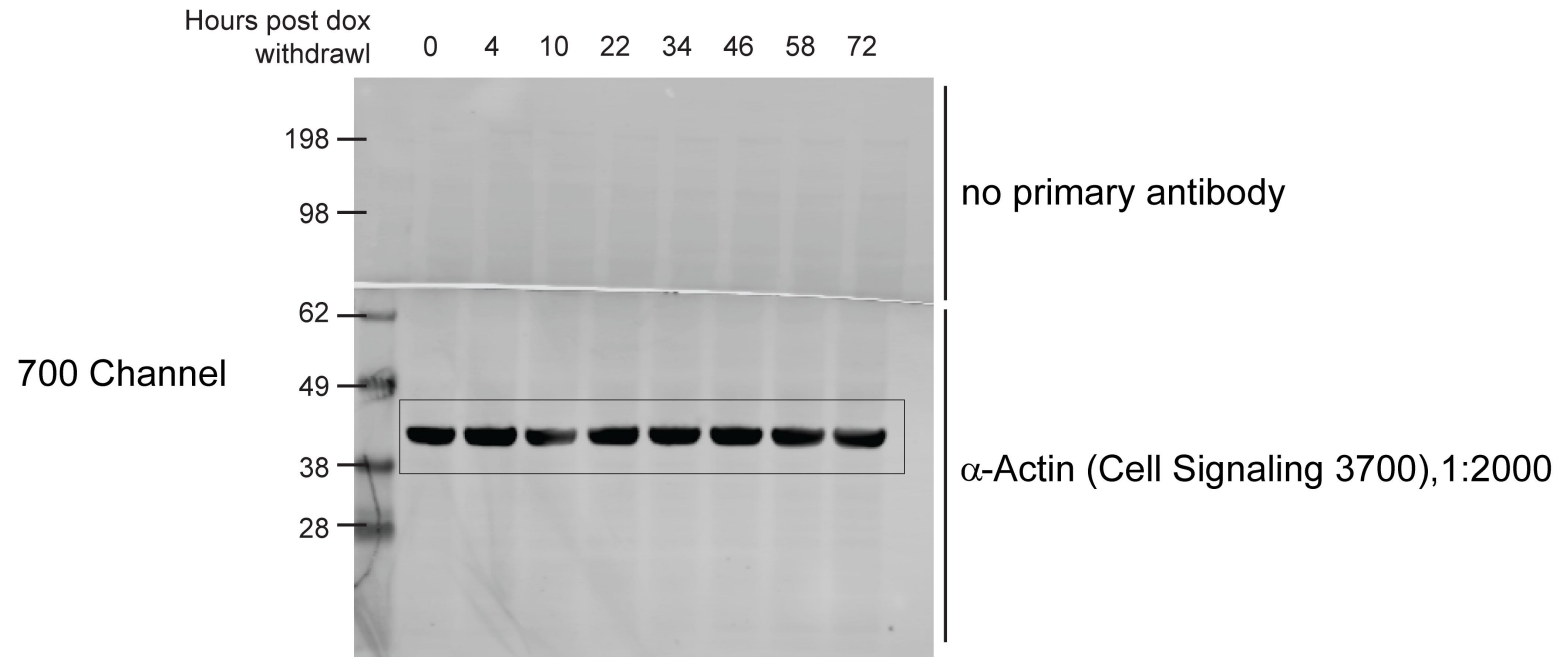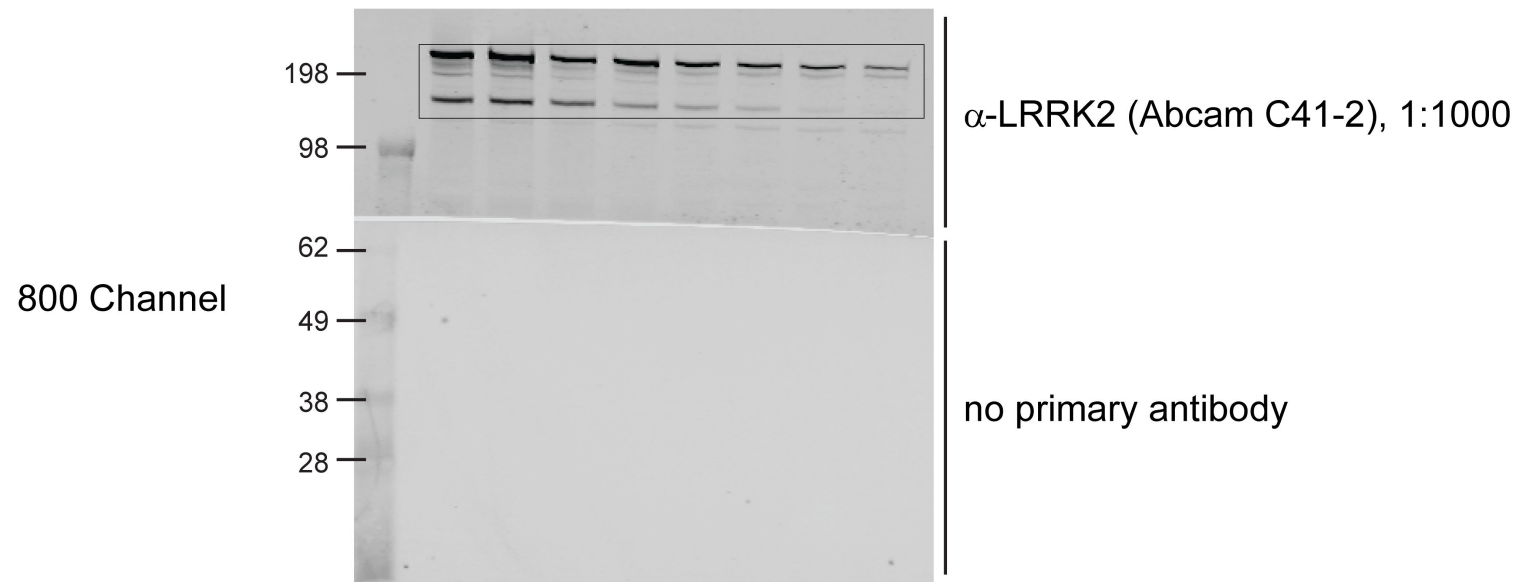

Figure S2e Source Data

Blot 1

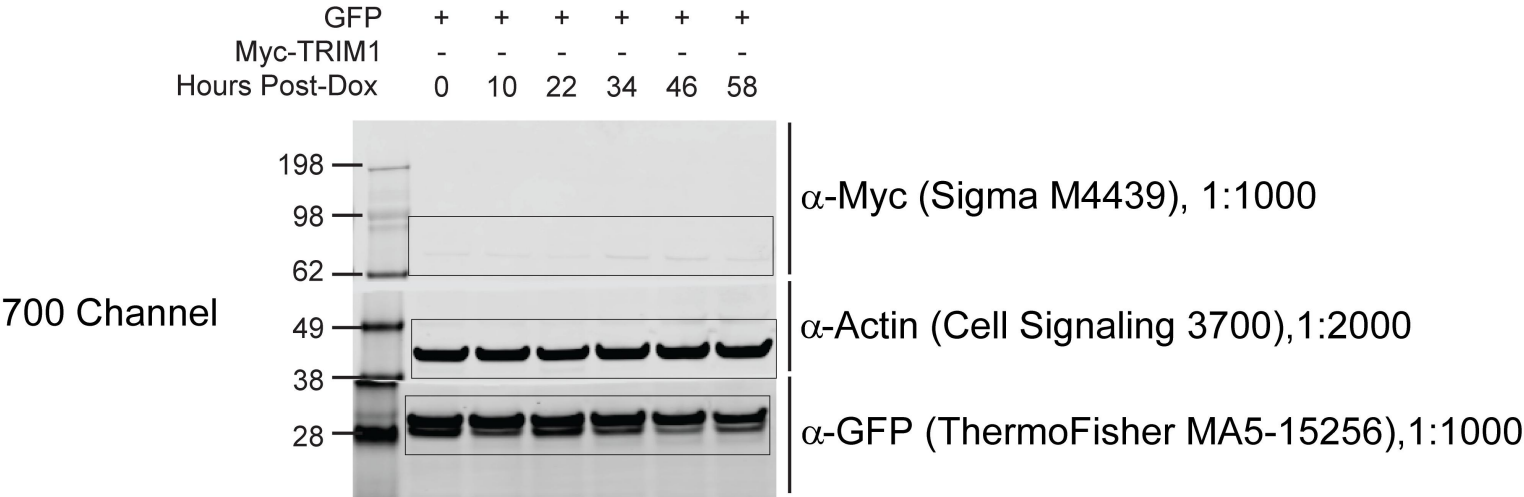

Blot 2

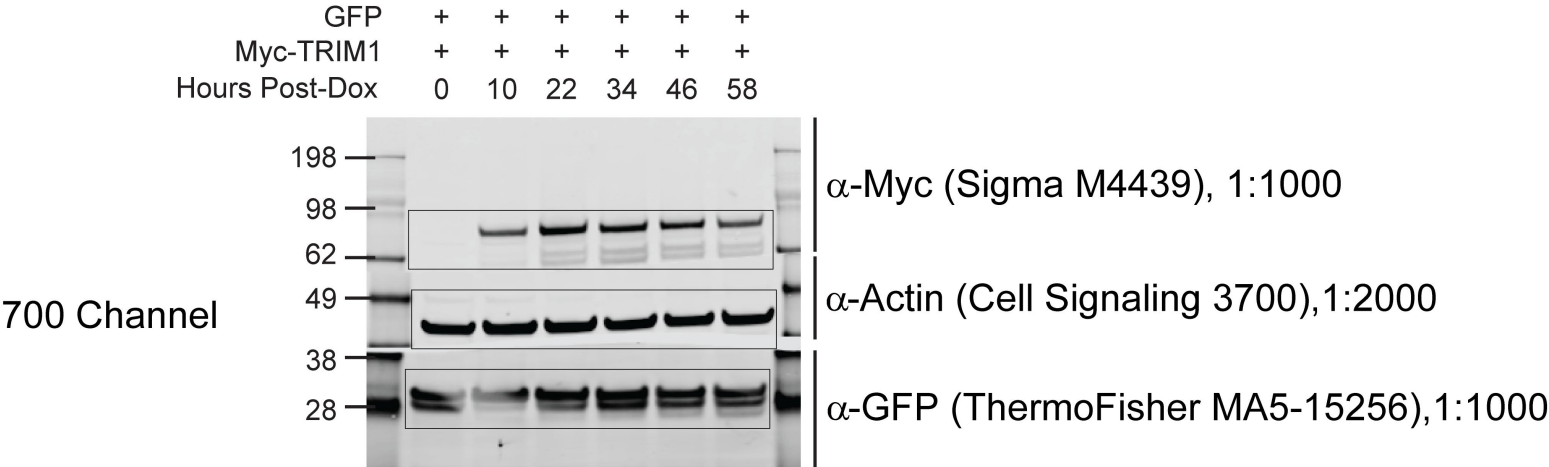

Supplement: SourceData FS2 — is the source file for Fig. S2. [file JCB_202010065_SourceDataFS2.pdf]

Supplemental Figure S5c

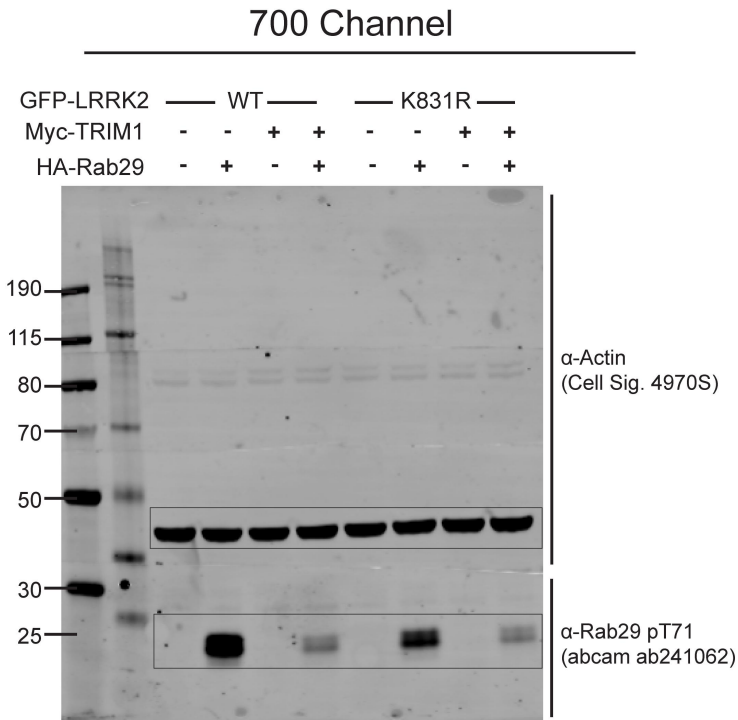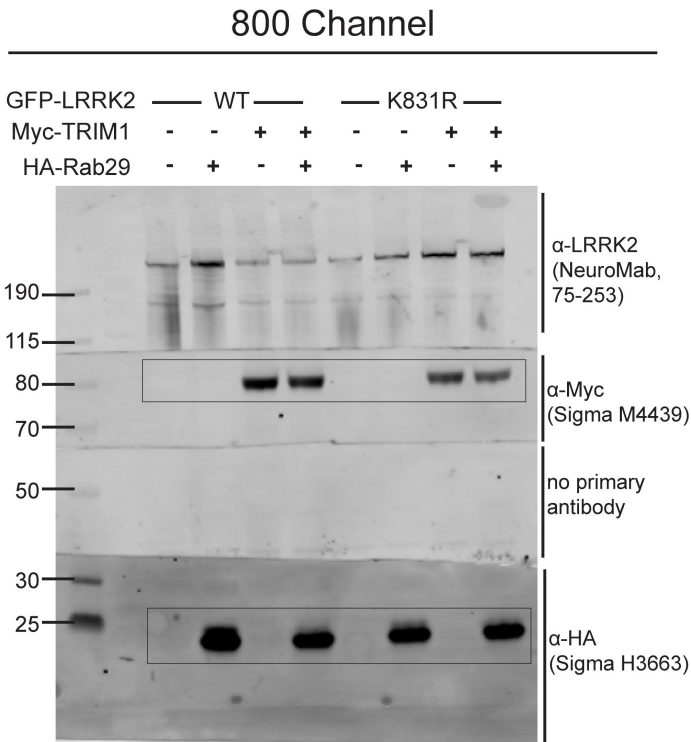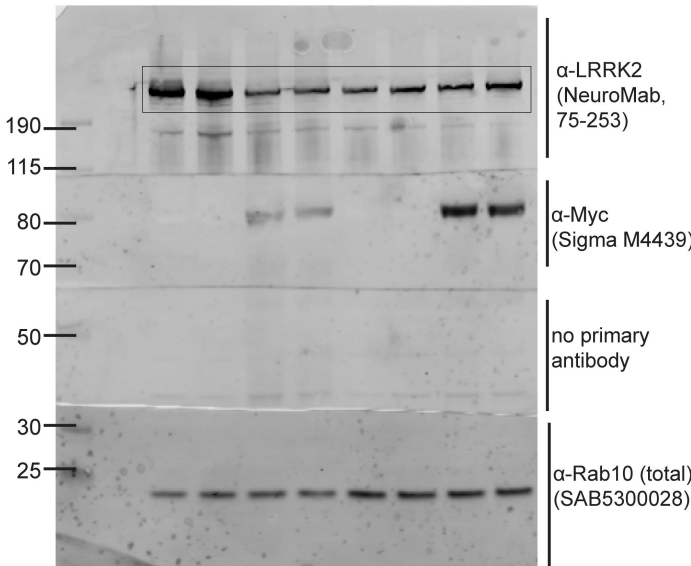

Supplemental Figure S5f

700 Channel

800 Channel

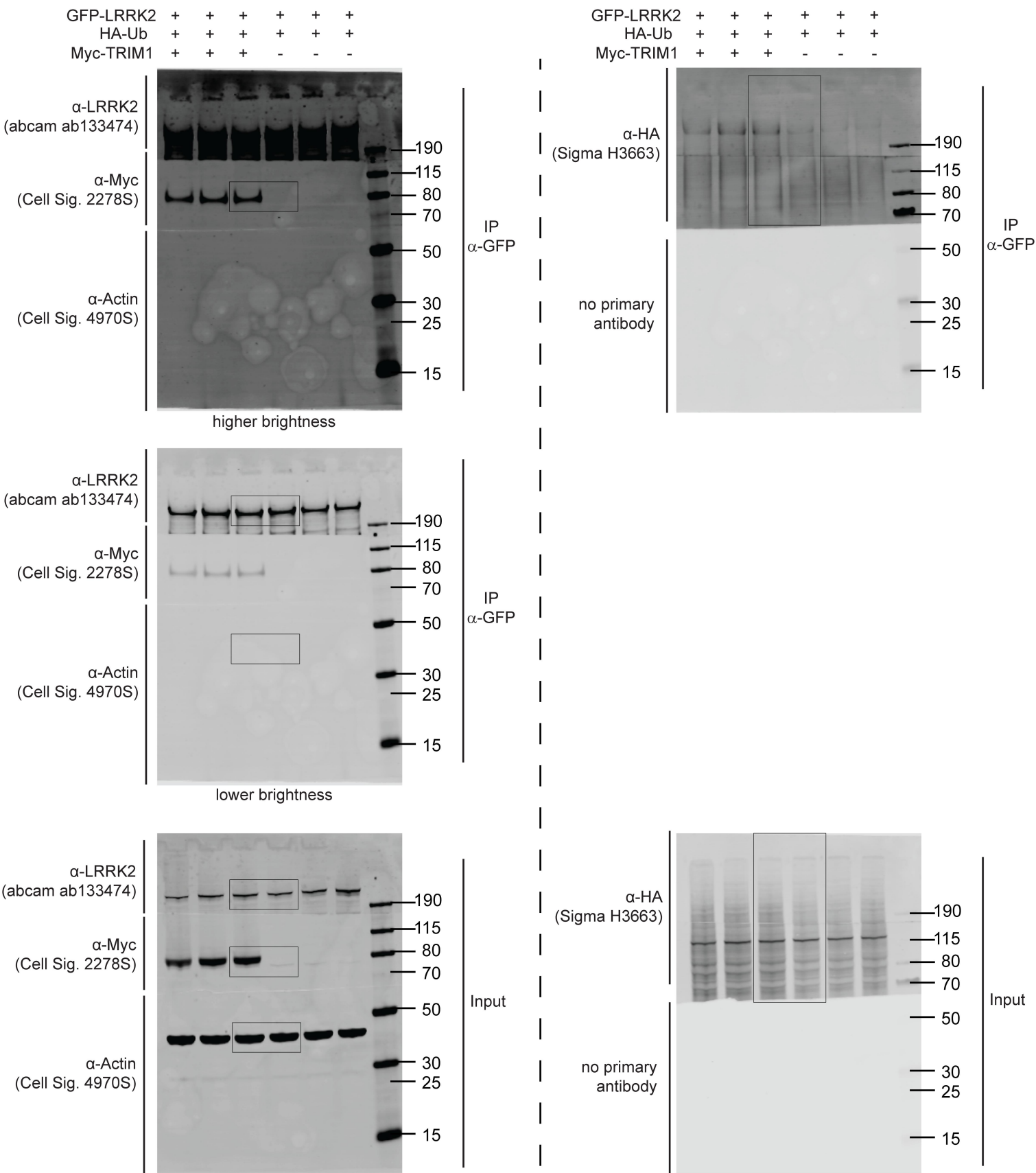

Supplement: SourceData FS5 — is the source file for Fig. S5. [file JCB_202010065_SourceDataFS5.pdf]
