# Supplementary material for: The E3 ligase TRIM1 ubiquitinates LRRK2 and controls its localization, degradation, and toxicity
Source: J Cell Biol. 2022 Mar 10;221(4):e202010065. doi: 10.1083/jcb.202010065 (PMC8919618; doi:10.1083/jcb.202010065)

Figure 5a Source Data

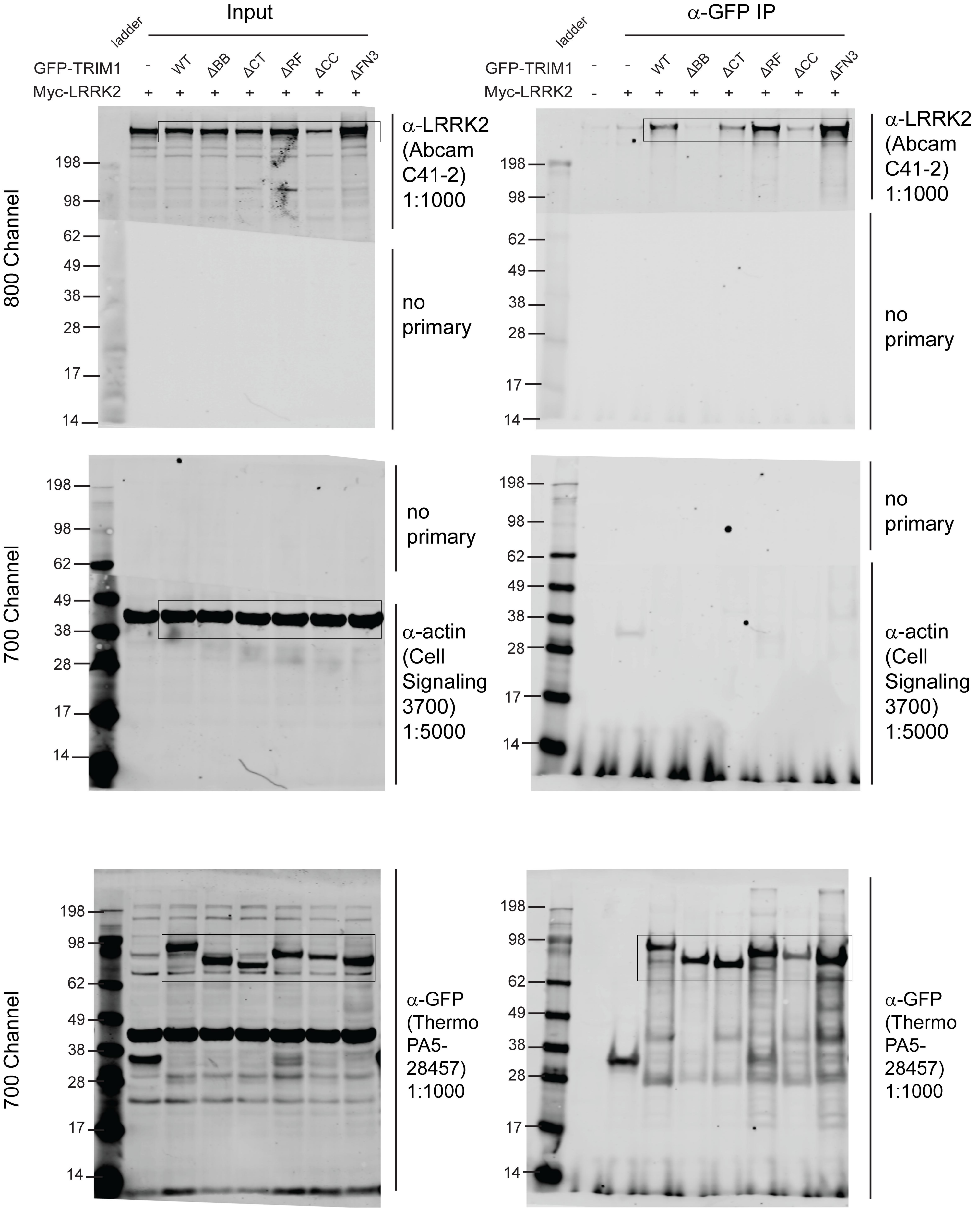

Figure 5b Source Data

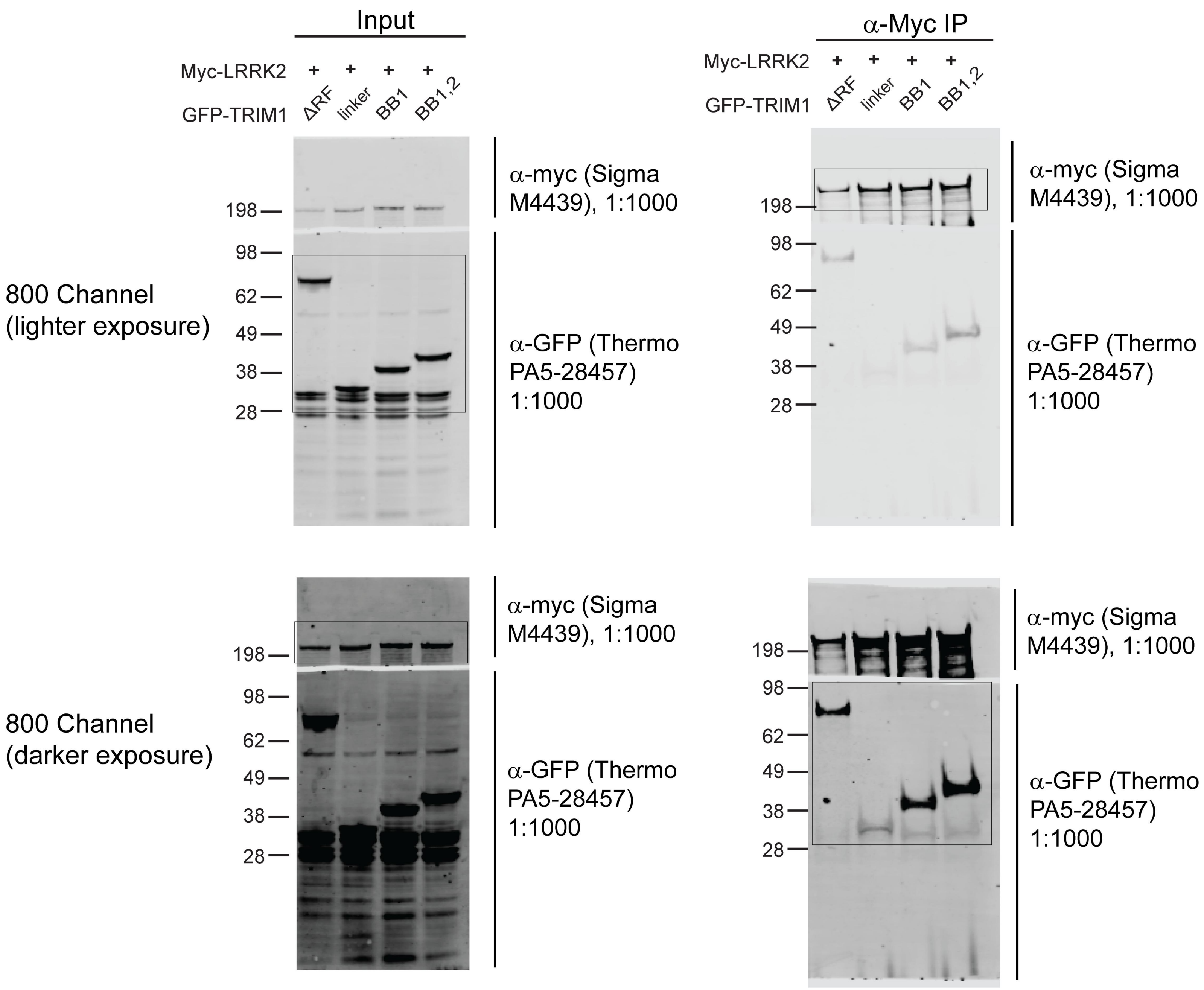

Figure 5c Source Data

Blot 1

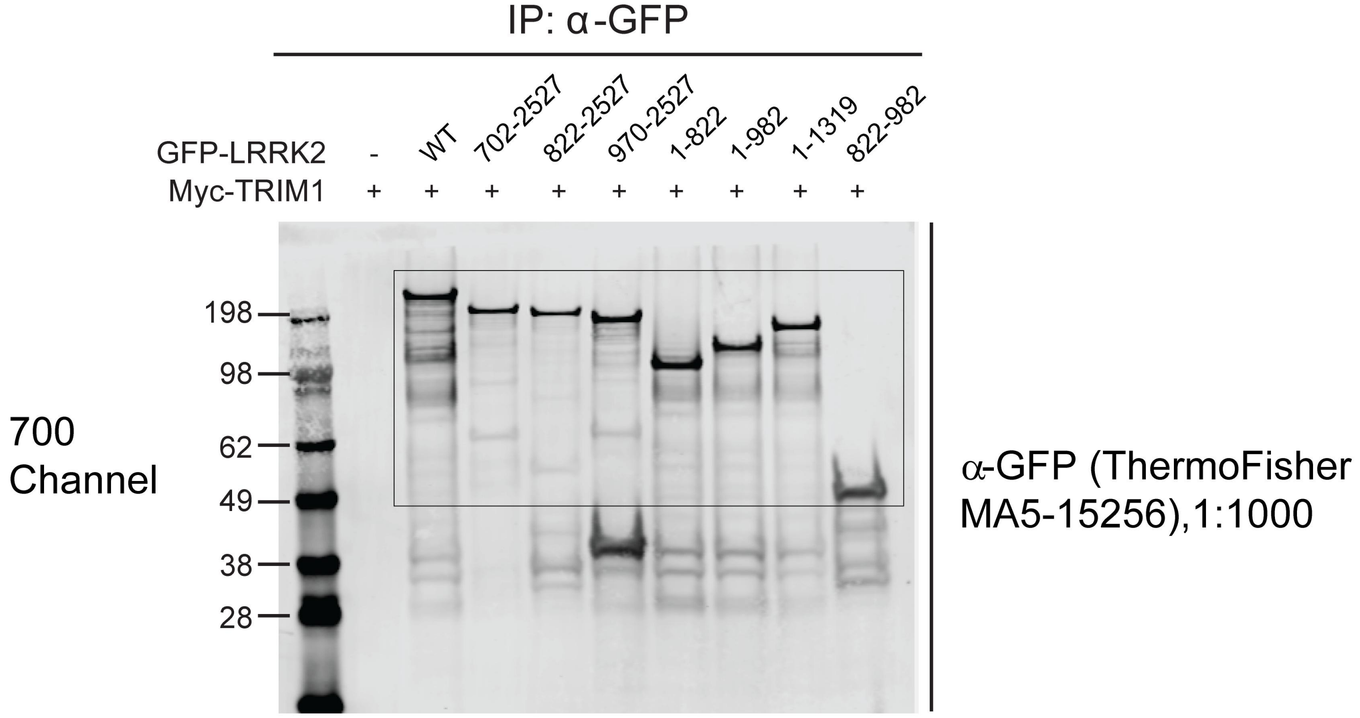

Blot 2

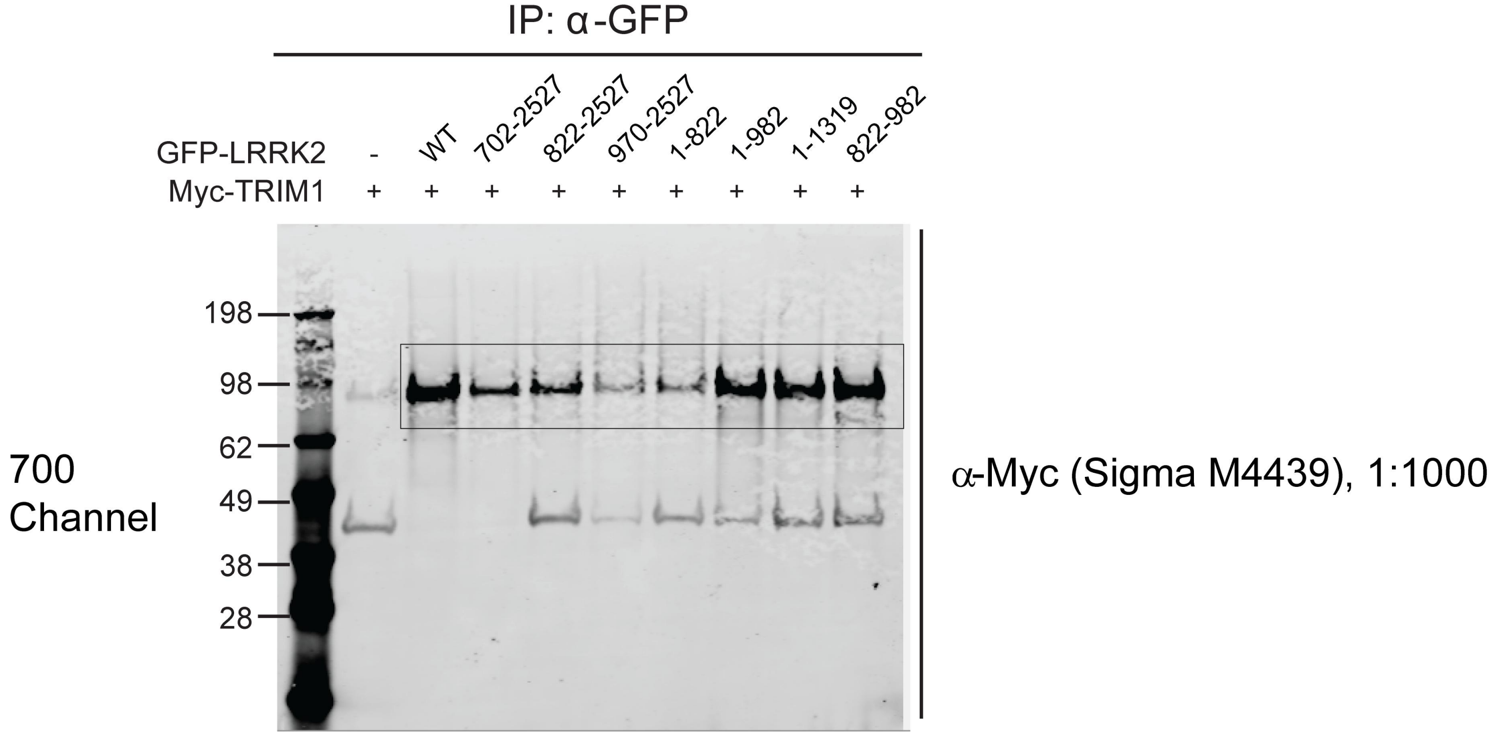

Figure 5c Source Data

Blot 3

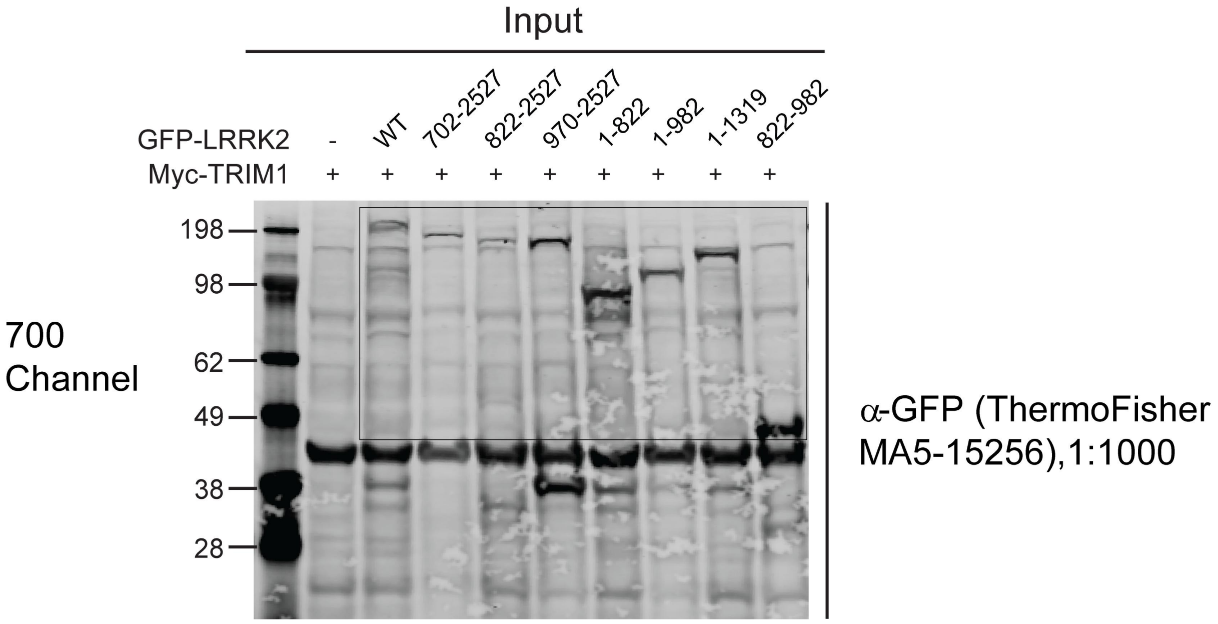

Blot 4

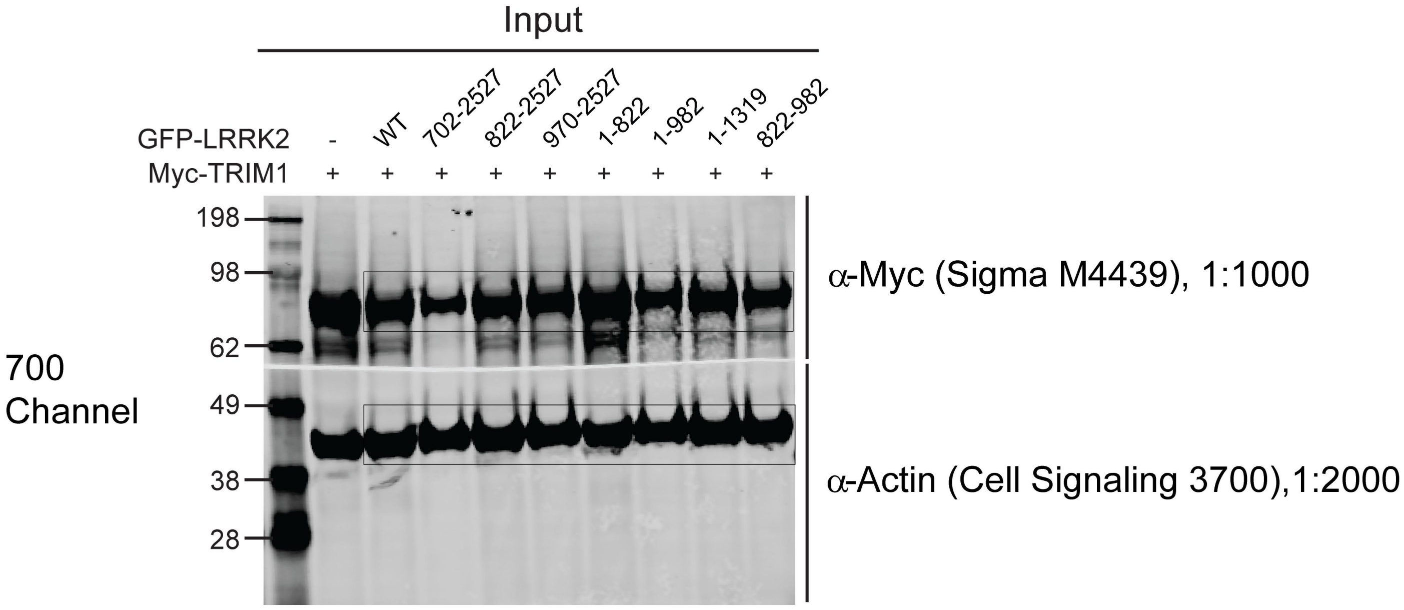

Figure 5f Source Data

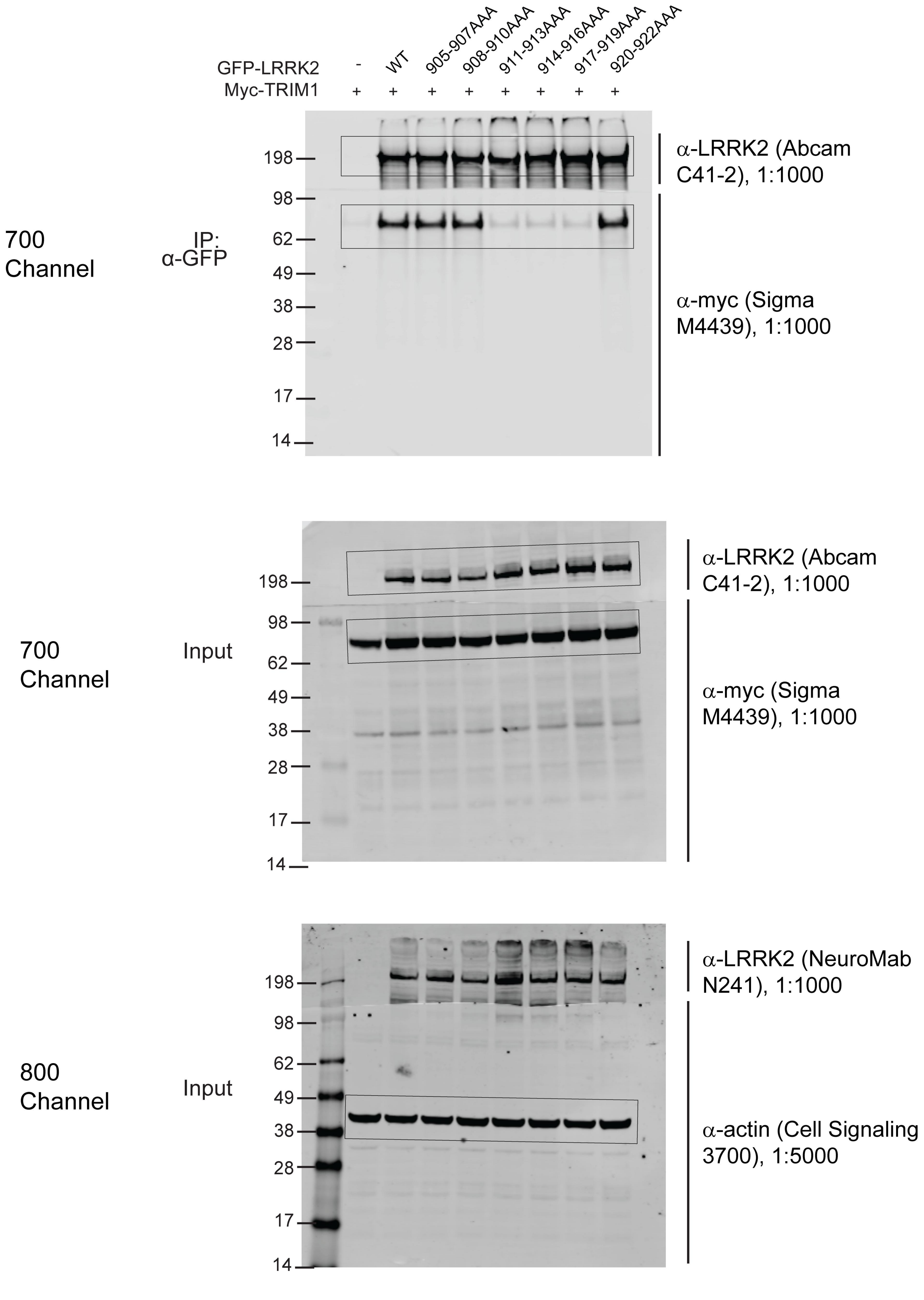

Figure 5h_page1

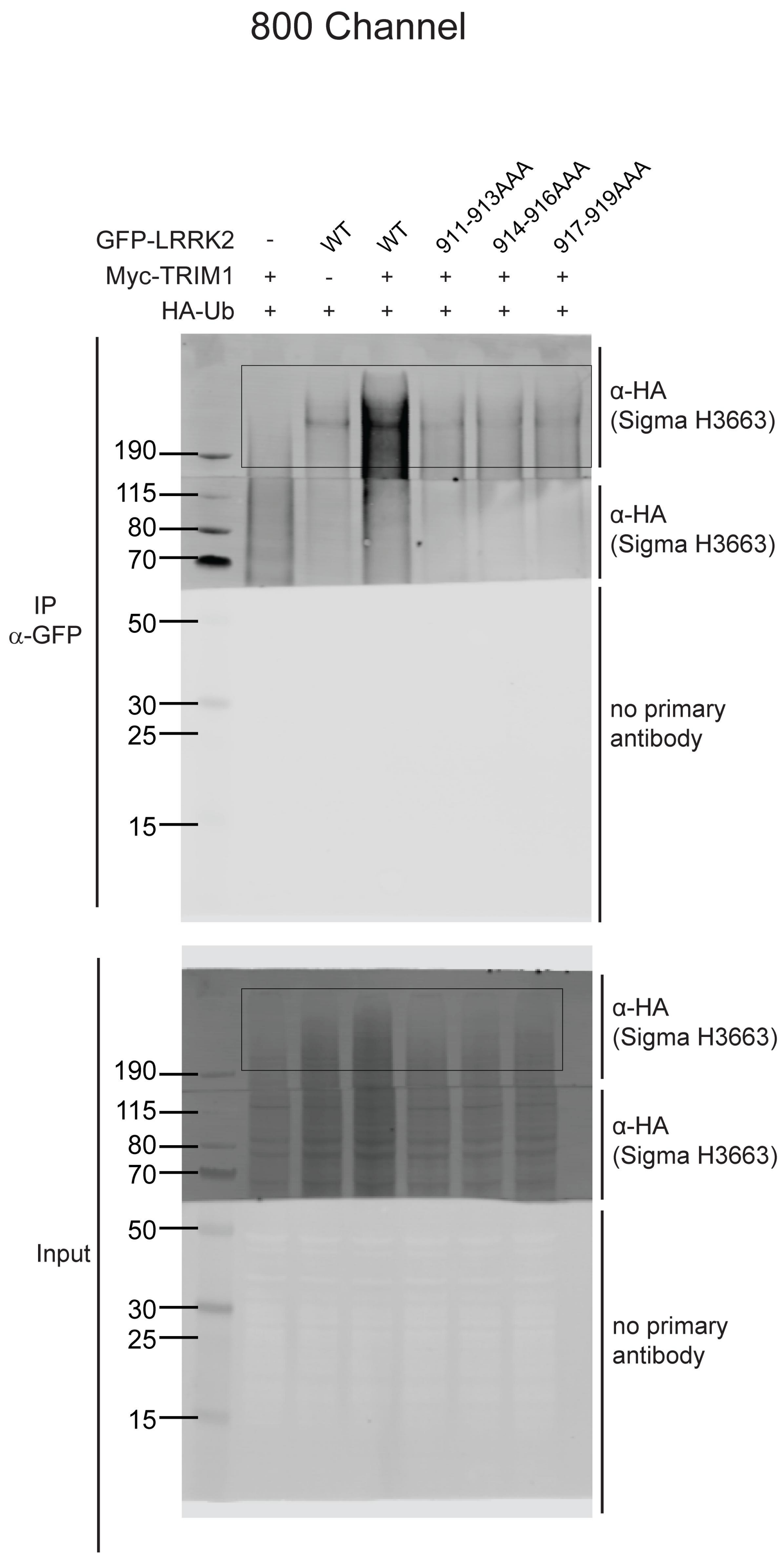

Figure 5h_page2

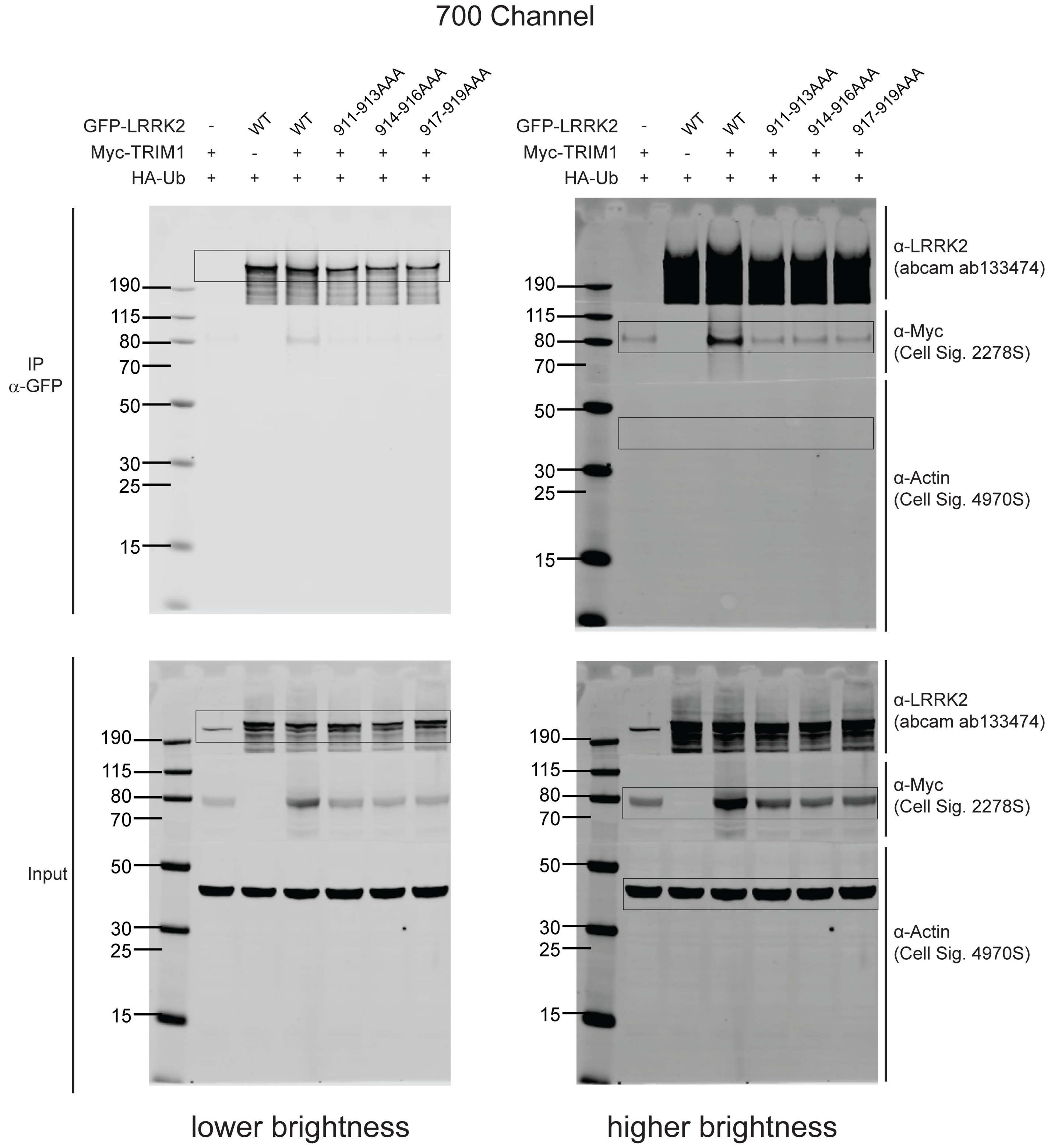

Supplement: SourceData F5 — is the source file for Fig. 5. [file JCB_202010065_SourceDataF5.pdf]
