# Supplementary material for: The E3 ligase TRIM1 ubiquitinates LRRK2 and controls its localization, degradation, and toxicity
Source: J Cell Biol. 2022 Mar 10;221(4):e202010065. doi: 10.1083/jcb.202010065 (PMC8919618; doi:10.1083/jcb.202010065)

Source Data Figure 9b

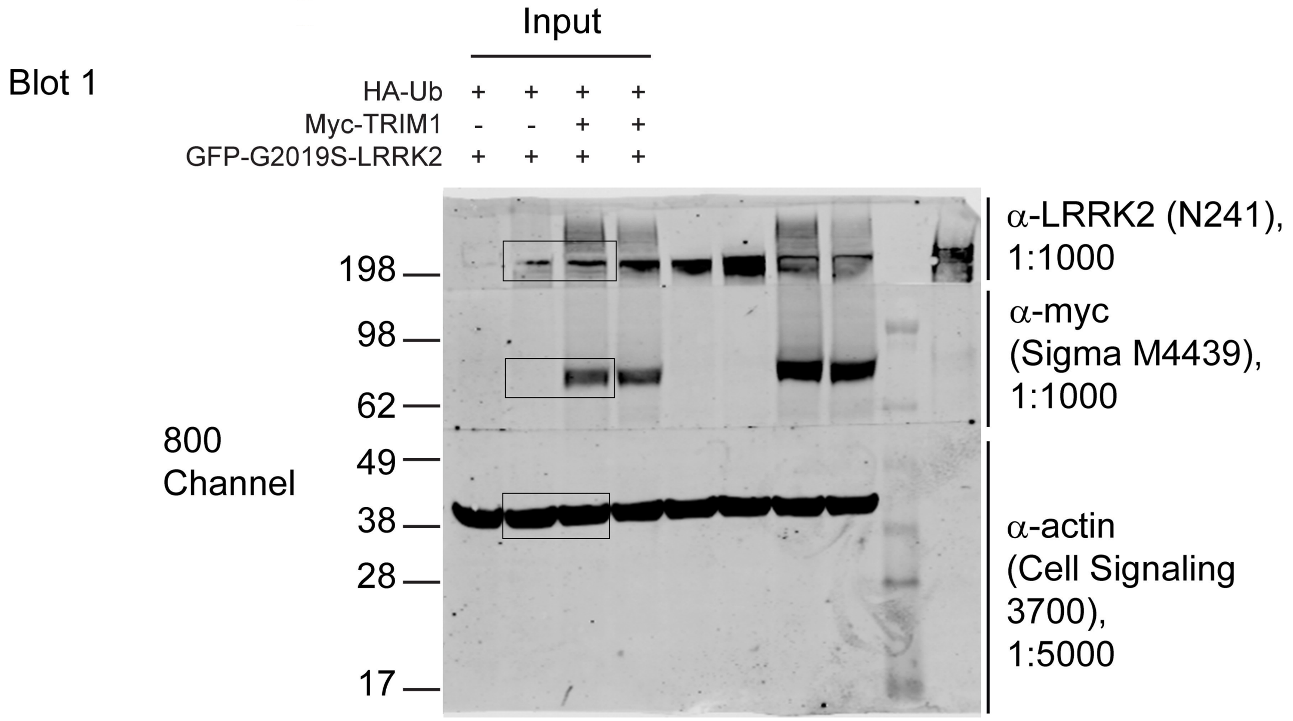

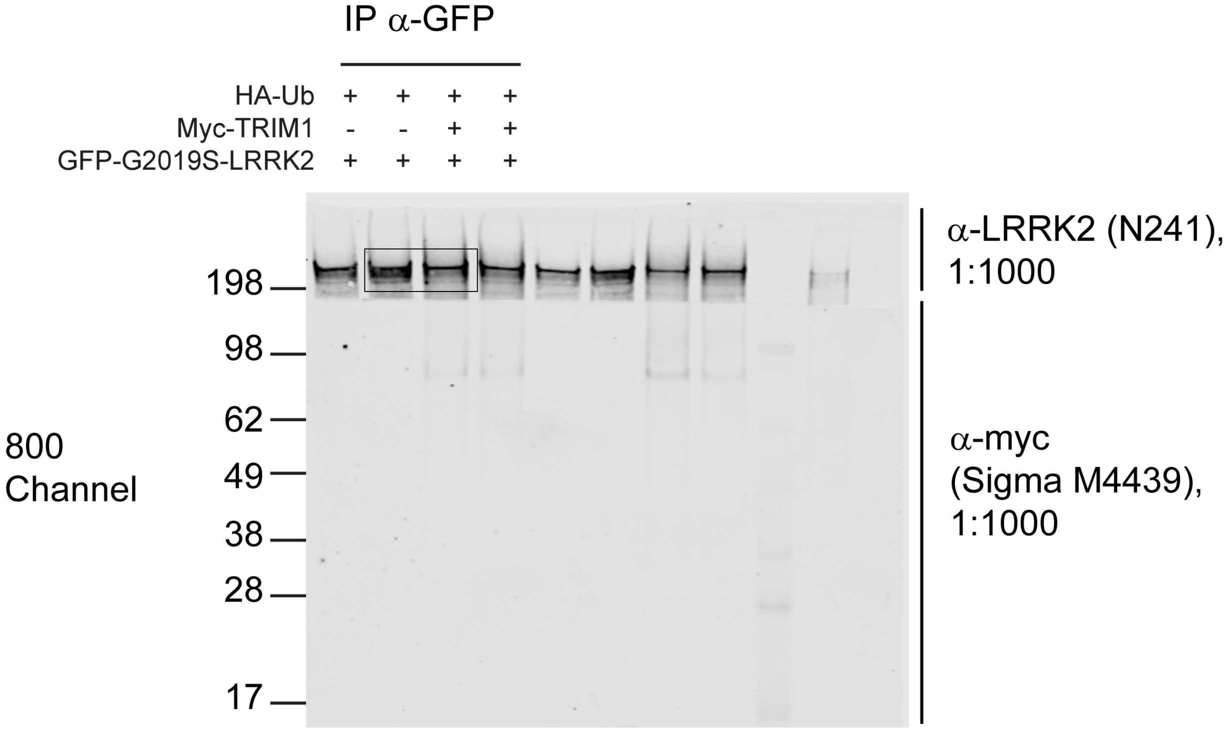

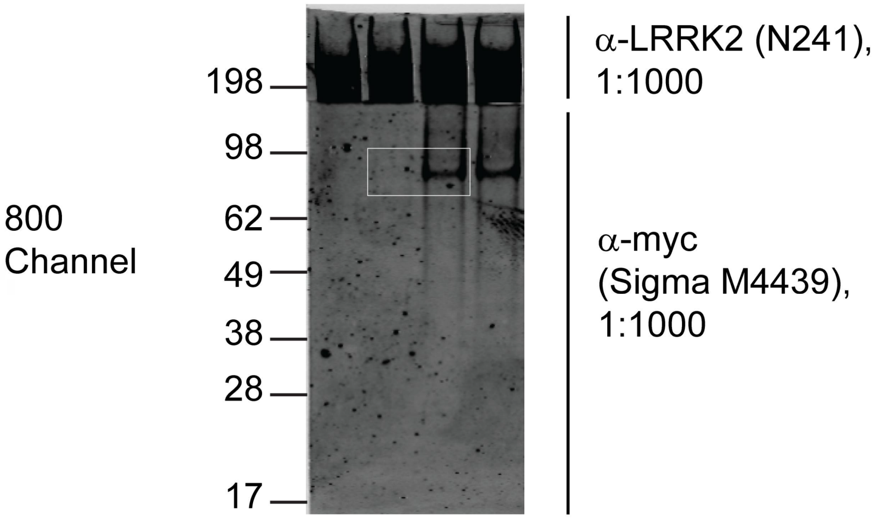

Source Data Figure 9b

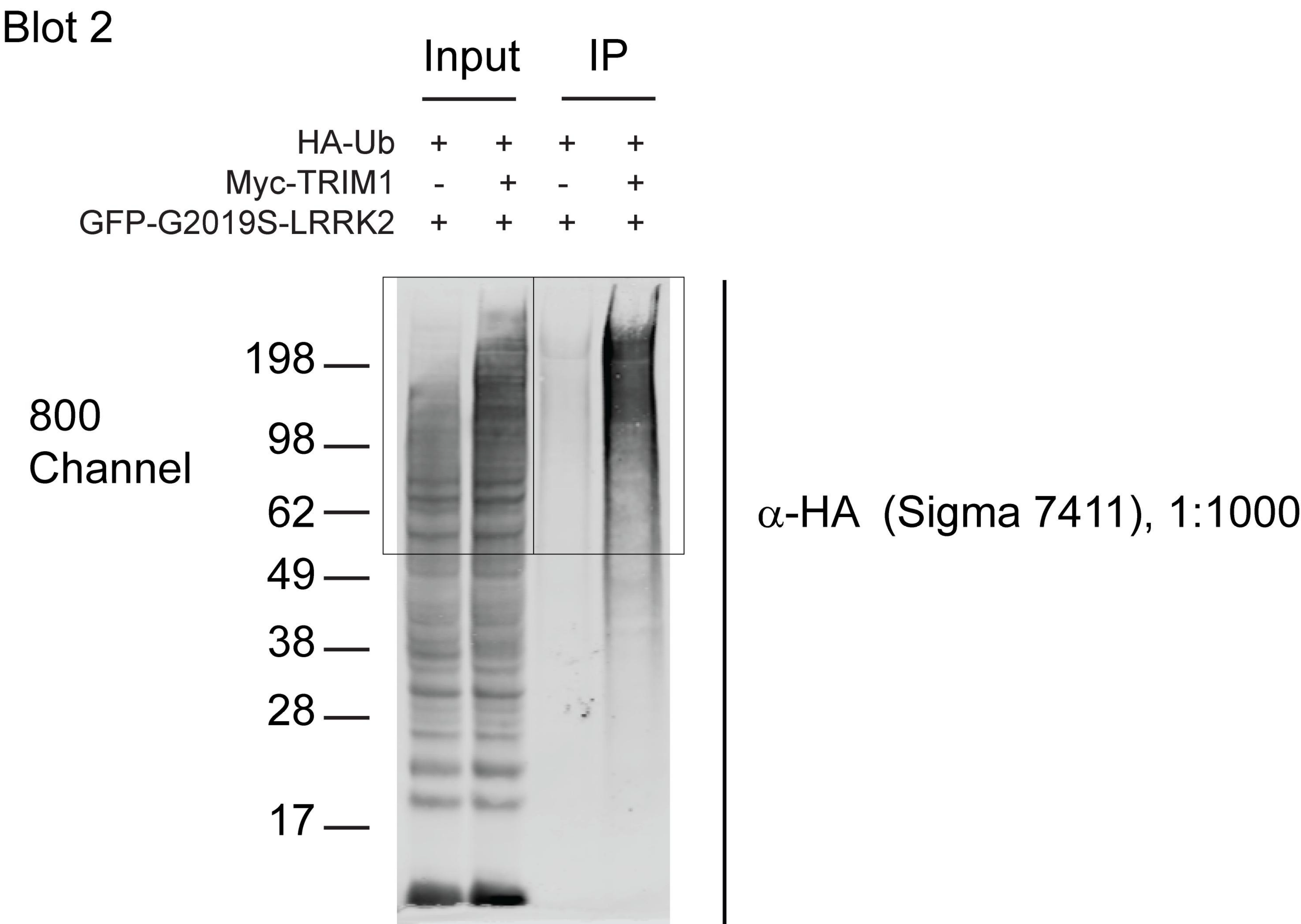

Supplement: SourceData F9 — is the source file for Fig. 9. [file JCB_202010065_SourceDataF9.pdf]
