# Supplementary material for: The E3 ligase TRIM1 ubiquitinates LRRK2 and controls its localization, degradation, and toxicity
Source: J Cell Biol. 2022 Mar 10;221(4):e202010065. doi: 10.1083/jcb.202010065 (PMC8919618; doi:10.1083/jcb.202010065)

Source Data Figure S3b

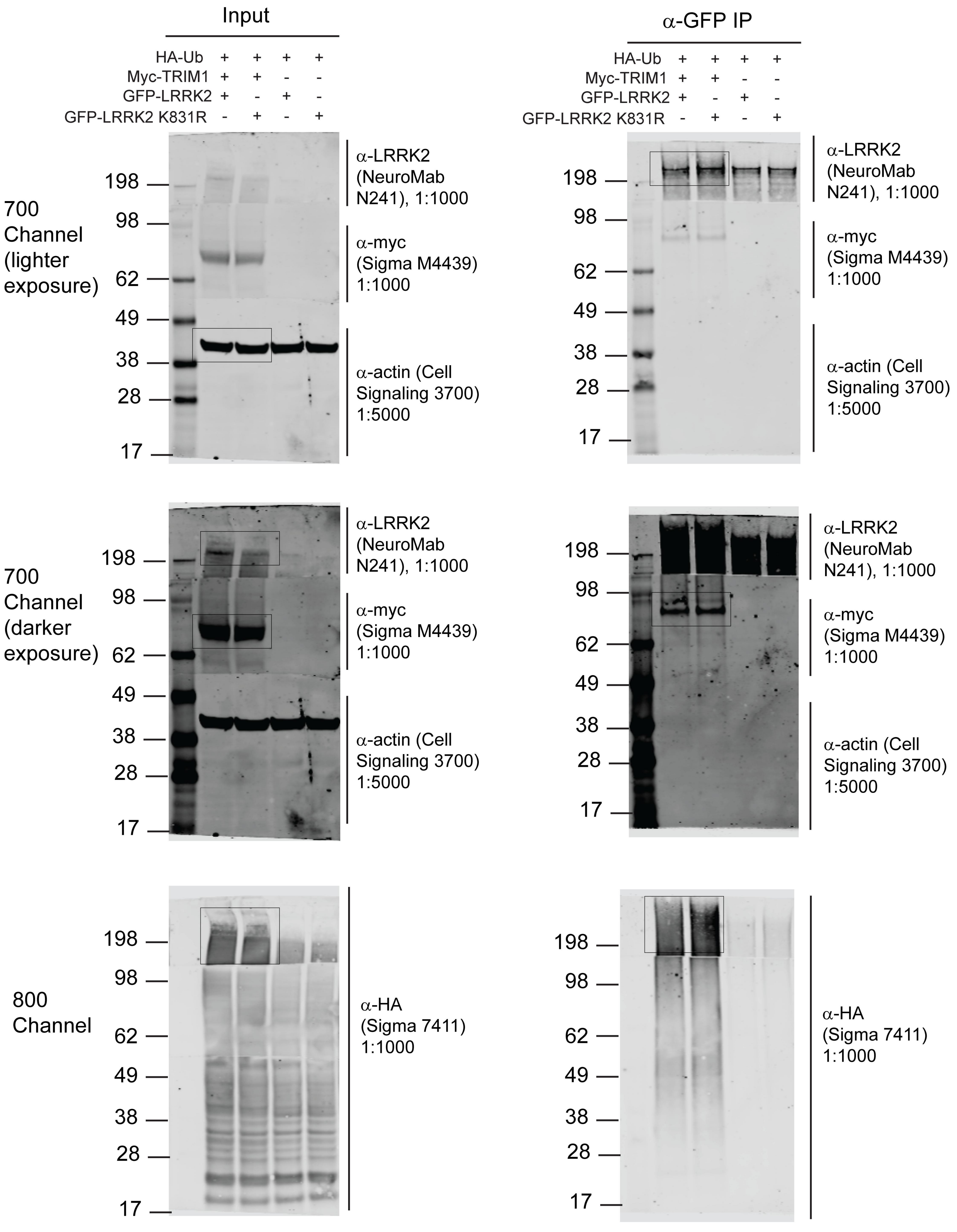

Supplemental Figure S3d_page1

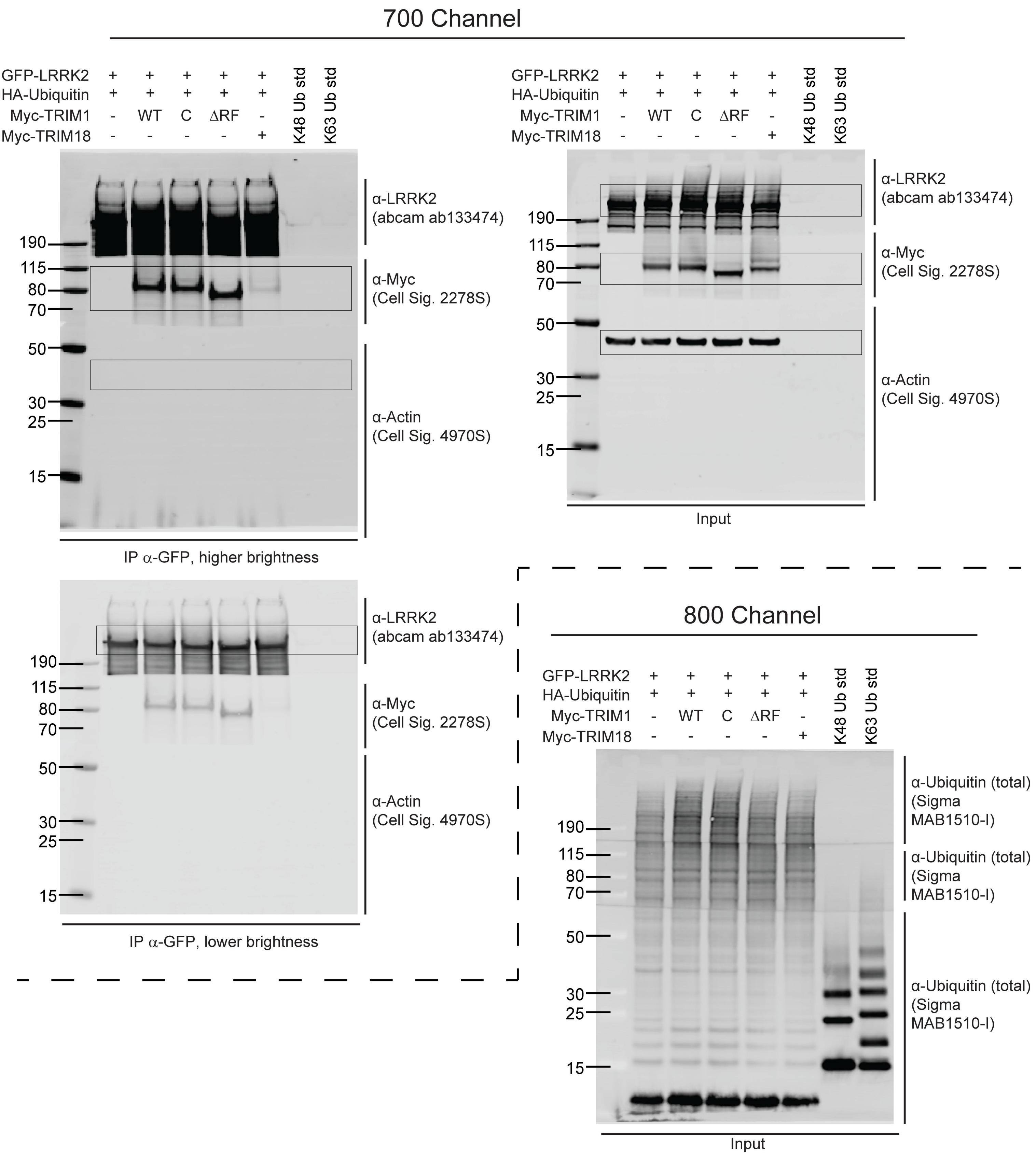

Supplemental Figure S3d_page2

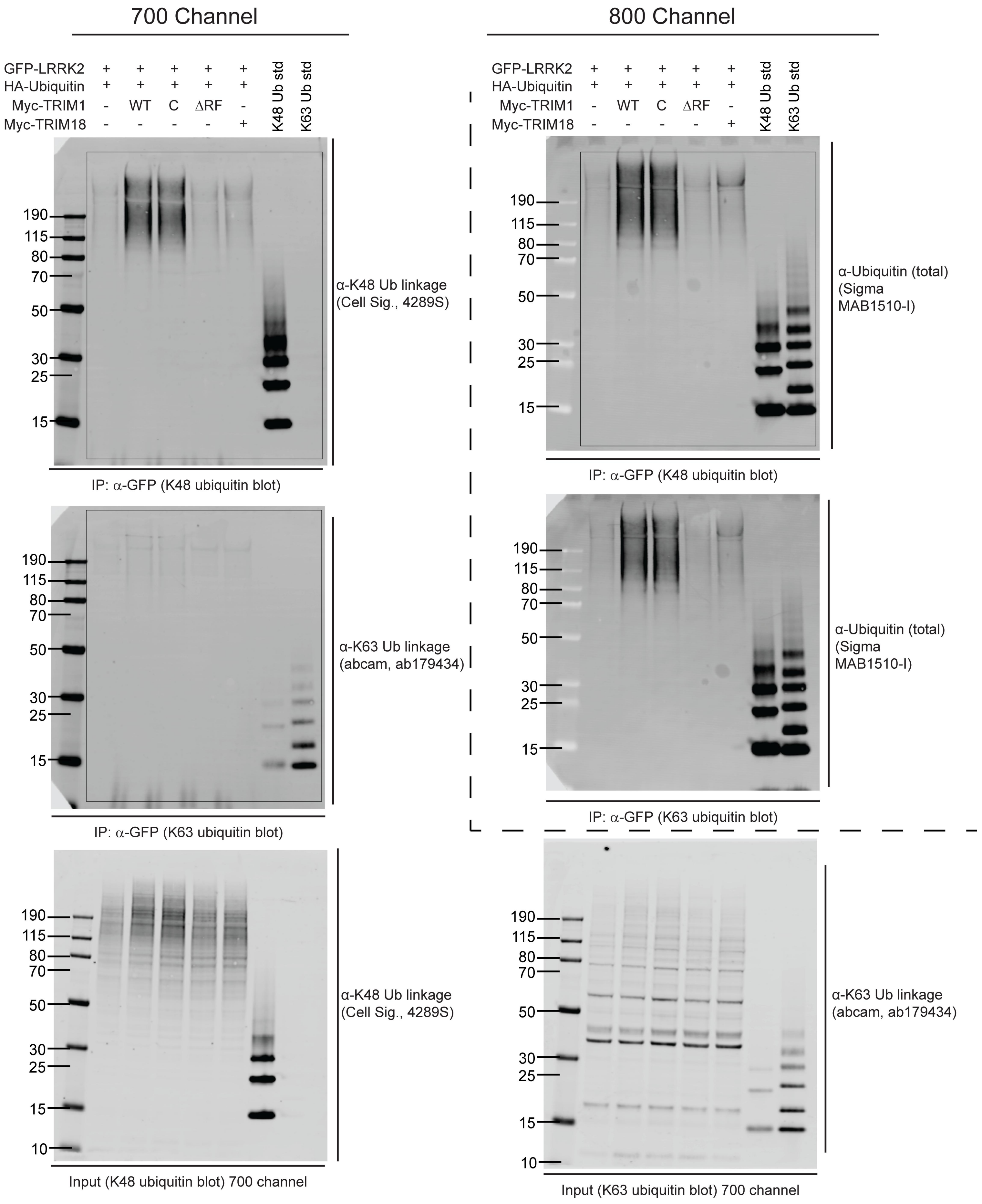

Source Data FS3f_page1

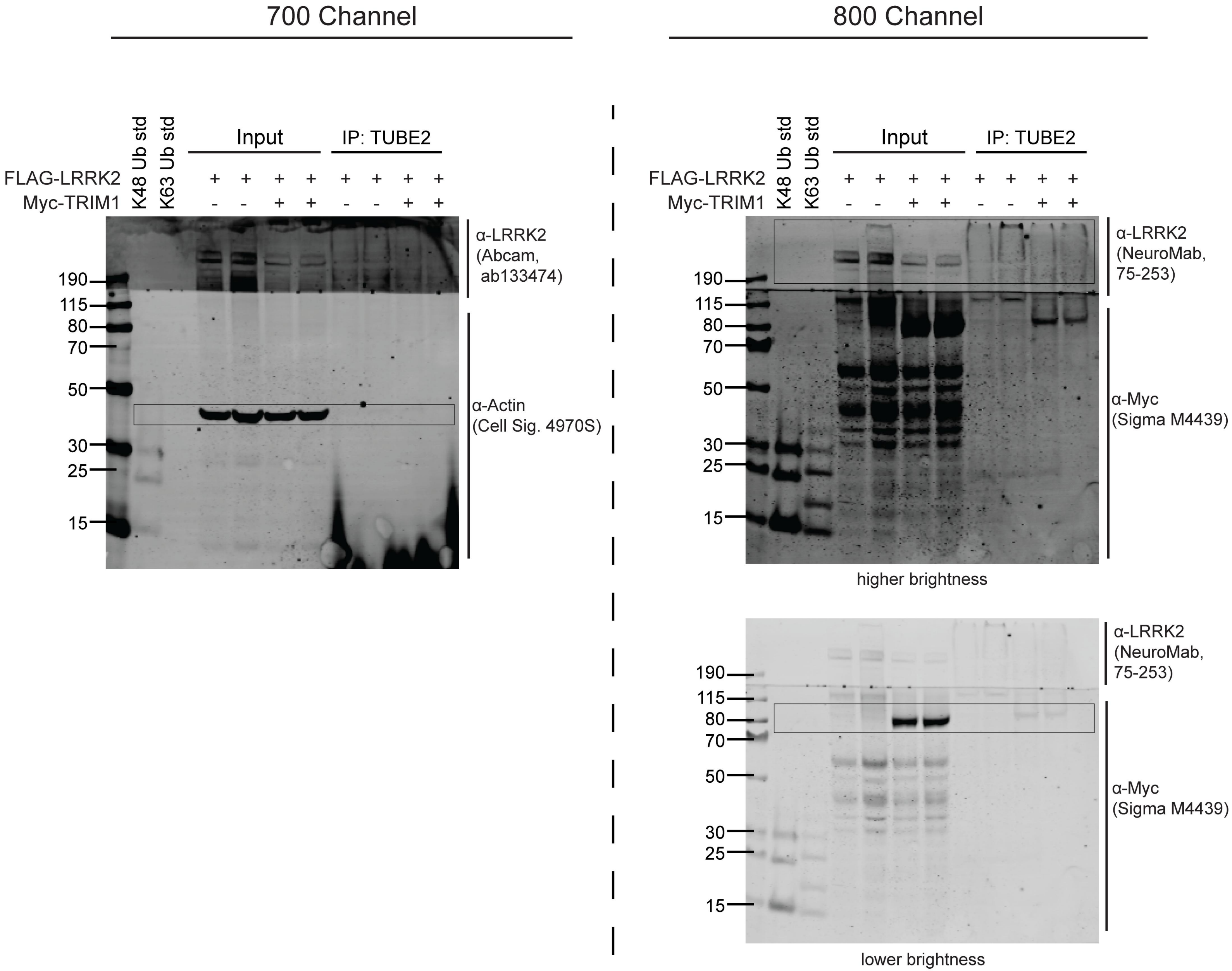

Source Data FS3f_page2

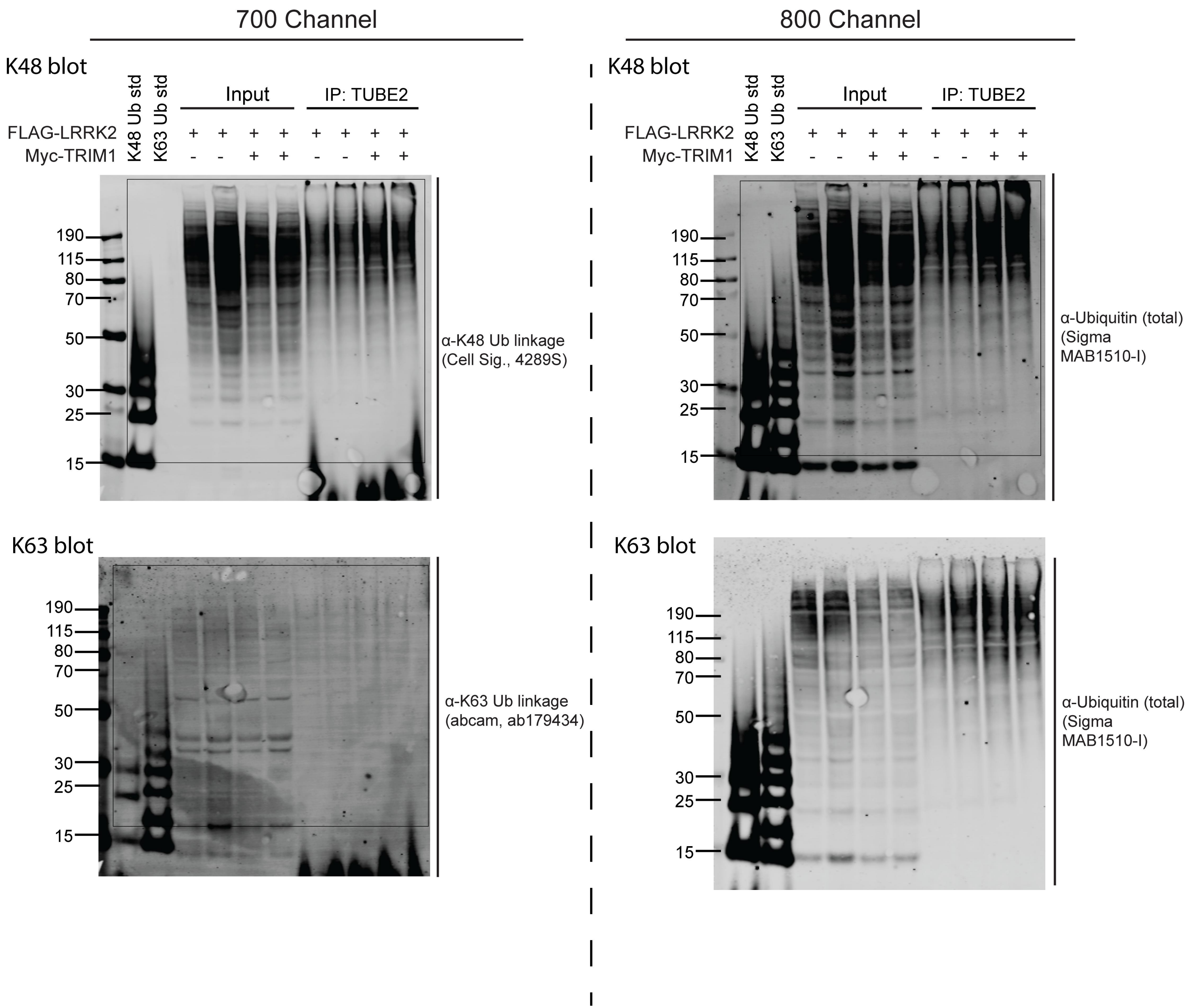

Supplement: SourceData FS3 — is the source file for Fig. S3. [file JCB_202010065_SourceDataFS3.pdf]
